# Development of a Novel Microwave Distillation Technique for the Isolation of *Cannabis sativa* L. Essential Oil and Gas Chromatography Analyses for the Comprehensive Characterization of Terpenes and Terpenoids, Including Their Enantio-Distribution

**DOI:** 10.3390/molecules26061588

**Published:** 2021-03-13

**Authors:** Giuseppe Micalizzi, Filippo Alibrando, Federica Vento, Emanuela Trovato, Mariosimone Zoccali, Paolo Guarnaccia, Paola Dugo, Luigi Mondello

**Affiliations:** 1 Chromaleont s.r.l., c/o Department of Chemical, Biological, Pharmaceutical and Environmental Sciences, University of Messina, Viale Palatucci, Polo Universitario Annunziata, 98168 Messina, Italy; filippo.alibrando@chromaleont.it (F.A.); federica.vento@chromaleont.it (F.V.); emanuela.trovato@chromaleont.it (E.T.); pdugo@unime.it (P.D.); lmondello@unime.it (L.M.); 2 Department of Mathematical and Computer Science, Physical Sciences and Earth Sciences, University of Messina, Viale Ferdinando Stagno d’Alcontres 31, 98166 Messina, Italy; mzoccali@unime.it; 3 Department of Agriculture, Food and Environment (Di3A), University of Catania, via Valdisavoia 5, 95123 Catania, Italy; paolo.guarnaccia@unict.it; 4 Department of Chemical, Biological, Pharmaceutical and Environmental Sciences, University of Messina, Viale Palatucci, Polo Universitario Annunziata, 98168 Messina, Italy; 5 BeSep s.r.l., c/o Department of Chemical, Biological, Pharmaceutical and Environmental Sciences, University of Messina, Viale Palatucci, Polo Universitario Annunziata, 98168 Messina, Italy

**Keywords:** cannabis EO, hemp, microwave hydro-distillation, GC-MS/FID, enantio-GC

## Abstract

A microwave distillation method was optimized for the extraction and isolation of cannabis essential oil from fresh and dried hemp inflorescences. The developed method enabled us to obtain a distilled product rich in terpenes and terpenoid compounds, responsible of the typical and unique smell of the cannabis plant. The distillate from different hemp cultivars, including Kompolti, Futura 75, Carmagnola, Felina 32 and Finola were characterized by using a gas chromatograph equipped with both mass spectrometer and flame ionization detectors. In a single chromatographic run, the identity and absolute amounts of distilled compounds were determined. Peak assignment was established using a reliable approach based on the usage of two identification parameters, named reverse match, and linear retention index filter. Absolute quantification (mg g^−1^) of the analytes was performed using an internal standard method applying the flame ionization detector (FID) response factors according to each chemical family. An enantio-GC-MS method was also developed in order to evaluate the enantiomeric distribution of chiral compounds, an analytical approach commonly utilized for establishing the authenticity of suspicious samples.

## 1. Introduction

*Cannabis Sativa* L. has gained much attention over the last decades due to the ability of this plant to biosynthesize phytocannabinoids, a class of terpenophenolic compounds whit a well-known pharmacological activity. Undoubtedly, the enormous interest around these compounds plays a fundamental role in the development of medical cannabis preparation [1]. Cannabis essential oils (EOs) have been attracting more and more attention from different industries. The typical and unique smell of this plant generates a particular interest around the global flavor and fragrance market, for which a detailed study on hydrocarbon terpenes and oxygenated derivatives in cannabis EO was necessary. In 1999, the European Commission’s Scientific Committee on Cosmetic Products and Non-Food Products (SCCNFP) identified a set of fragrance allergens with a well-recognized potential to cause contact allergy in susceptible individuals [2]. In such respect, the *European Directive 2003/15/EC* reported the cosmetic irritant list, for which their presence must be specified in the ingredient list of cosmetic products in order to avoid allergic reactions in consumers. Consequently, it becomes crucial to also analyze the terpene content in cannabis EO with the aim to provide detailed information in terms of quality and safety, especially in cosmetic fields, considering that cannabis EO is used in cream, ointment, gel, or applied over the skin [3].

Data in the literature have also highlighted a probable contribution of terpenes on the pharmaceutical properties of cannabis-based medical products, defining the synergic action of terpenes and cannabinoids as entourage effects [4]. In such respect, α-pinene has been described as an acetylcholinesterase inhibitor that counteracts the Δ9-tetrahydrocannabinol (δ^9^-THC) intoxicant effect [5]. Myrcene is one of the predominant monoterpene hydrocarbons detected in numerous cannabis varieties and it is believed to be responsible for the narcotic-like sedative effects of several cannabis preparations [3]. Additionally, (*E*)-caryophyllene, which represents the most abundant sesquiterpene hydrocarbon, interacts with the cannabinoid receptor type 2 and is responsible for the anti-inflammatory effects of some cannabis-based preparations [5]. Thus, apart from the aromatic properties, these compounds also show relevant therapeutic effects that emphasize the importance to investigate the terpene content of cannabis plants, especially those characterizing medical chemotypes used for therapeutic purposes [4].

From the botanical point of view, *Cannabis Sativa* L. is a dioecious, rarely monoecious, annual flowering plant, member of the *Cannabaceae* family [6]. Female inflorescences are densely covered with glandular trichomes containing resin, one of the most valuable cannabis products with its various psychoactive and medicinal properties [7]. The resin contains secondary metabolites including terpene molecules. Over 200 terpenes have already been identified in the flowers and leaves of the cannabis plant, which may represent up to 10% of the total trichome content [8]. They are repellent chemical constituents that the plant utilizes as defensive strategy against many insects [9]. The terpene fingerprint is a phenotypic trait that shows high variability both across different cannabis cultivars and between specimens of the same variety exposed to different environmental conditions [9]. For example, it has been demonstrated that the quantity of terpenes increases with light exposition and decreases with soil fertility [7]. In addition, the position of the inflorescence along the flowering stream influences the characteristic of the terpene profiles, according to the light exposition projected from above [10]. All these factors highlight arduous work of the analysts, complicated even by the fact that over the time numerous botanical varieties and different genetic manipulations have been produced. Nowadays, approximately 700 cultivars have ready been described [11].

Data in the literature report numerous methodologies applied for the production of cannabis EO from inflorescences. In 2003, Romano and Hazekamp [12] provided an extensive research study in which they compared the extraction performances of different solvents including ethanol, naphtha, petroleum ether, and olive oil. The use of olive oil as an extraction solvent was found to be the most performant in term of terpene quantity extracted, probably due to its highly non-polar and non-volatile character, which guaranteed the correct solubilization of terpenes, avoiding their loss by evaporation [12]. Steam-distillation (SD) and hydro-distillation (HD) have been also utilized for the extraction and isolation of cannabis EO. In this regard, Fiorini et al. [3] evaluated the effectiveness of these two different distillation techniques. HD technique, performed using a mantle system equipped with Clevenger-type apparatus, was revealed to be more efficient in the sesquiterpenes and cannabinoids enrichment of cannabis EO. On the other hand, the SD approach provided a crude oil richer in monoterpenes than HD. Similar results were obtained also by Romano and Hazekamp [12]. Ternelli et al. [13] introduced an innovative microwave apparatus to perform the distillation of cannabis EO. Microwave-assisted hydro-distillation (MAHD) has also been successfully applied by Fiorini et al. [14]. In this research, the optimal operative conditions such as microwave irradiation power, temperature, and distillation time were carefully optimized, confirming the high effectiveness of the MAHD methodology.

Gas chromatography (GC) is the mandatory separation technique useful for accurate terpene and terpenoids analysis. When coupled to mass spectrometry (MS), the GC methodology reveals the correct identity of the components of a mixture, providing analytical results which are highly reliable. However, in the case of monoterpene or sesquiterpene chemical classes, the fragmentation in ion source produces undistinguishable MS spectra, and difficulties arise in peak identification. In order to support the identification of compounds, a linear retention index (LRI) system is commonly used nowadays. Utilizing the information on retention time and MS fragmentation facilitates the peak assignment, avoiding mistaken identifications. The quantification of volatile compounds is widely performed through the use of a flame ionization detector (FID), one of the most used devices in the flavor and fragrance field due to its low cost and simplicity [15]. Enantio-GC analysis of terpenes and oxygenated derivatives is one of the most common analytical techniques used in control laboratories for revealing an eventual adulteration or manipulation and guarantees the genuineness of an EO. In nature, the chirality presents a highly typical distribution of enantiomers resulting as a useful tool for the detection of human interference [16]. The enantiomeric distribution of terpenes and terpenoids is investigated by using a chiral GC column coated with a cyclodextrin-based stationary phase. To the best of the authors’ knowledge, only two manuscripts are reported in the literature for cannabis EO chiral investigation [8,14].

In light of the studies reported so far, the authors decided to develop a microwave-based distillation method for isolating cannabis EOs from five different hemp cultivars (Kompolti, Futura 75, Carmagnola, Felina 32 and Finola) and provide a detailed study of their volatile compositions. Terpenes, terpenoids, and cannabinoid substances were revealed using simultaneous MS and FID detections. In order to investigate the enantiomeric distribution in natural cannabis EOs, an enantio-GC-MS method was successfully optimized, and the trends of the most abundant optically active compounds are discussed in this manuscript.

## 2. Results and Discussion

### 2.1. Optimization of the Distillation Method

The aim of this study was to develop a solventless distillation protocol for obtaining good quality aromatic cannabis EO from different cultivars. For this purpose, a microwave-based distillation instrument, named Milestone ETHOS-X, was utilized. Microwave–water interactions cause an increase in the vibrational kinetic energy of molecules and results in the dispersion of heat to the vegetable material, and in particular to its oleiferous structures. Thus, heating induces the distension and stretching of the glandular trichome until the oleiferous glands break. Consequentially, an oil is extracted from the vegetable material, and a rapid evaporation of oil–water emulsion occurs. As reported in the literature [17], the distillation temperature is strictly connected not only to the type of plant and to the parts of the vegetal material, such as stems, leaves, flowers, or fruits, but also to the selected microwave power.

In the first part of this study, a moderate amount of time was spent on optimizing the distillation method. Microwave power, temperature, and distillation time were the parameters evaluated, with the aim to reach the highest yield of distillation without compromising the organoleptic properties of the cannabis EO. The optimization of the distillation method was carried out using a dried hemp variety of Futura 75. The first drops of EO were obtained after about 10 min of distillation at 1200 W, when a temperature of about 47 °C was registered in the glass reactor. After reaching the distillation temperature, the microwave power was reduced up to 700 W to avoid an excessive heating effect which could cause toasting of the inflorescences. Lower values of power (W) were also tested, but the distillation yields were not comparable with those obtained using 700 W. For a global evaluation of the distillation efficiency, four different time periods were tested, maintaining constant the microwave power at 700 W, including 10 min, 20 min, 30 min and 40 min. Longer time periods were not considered in this study due to the darker and certainly less-pleasant olfactive properties of cannabis oil. 

In term of percentage yield, the optimal conditions were obtained after 40 min of distillation time, counting a yield of 0.035% (*w*/*w*). Lower values of 0.024%, 0.025% and 0.019% were achieved after 30 min, 20 min and 10 min, respectively. Fresh inflorescences provided higher yields of cannabis EO than dried materials, which was in accordance with data reported in the literature [3]. Overall, Kompolti and Carmagnola were the fresh varieties more profitable in terms of oil production, producing yields of 0.274% and 0.210%, respectively. The obtained results were in good agreement with those published previously [14,18]. With respect to the dried inflorescences, the most abundant yields were obtained using Finola hemp inflorescences (0.109%). For a comprehensive evaluation, intra-day repeatability tests of the MAHD technique were also performed through analysis in triplicate of the same sample. The developed protocol showed a good data repeatability considering the relative standard deviation (RSD) of distillation yields of 16.63%.

**Figure 1:**
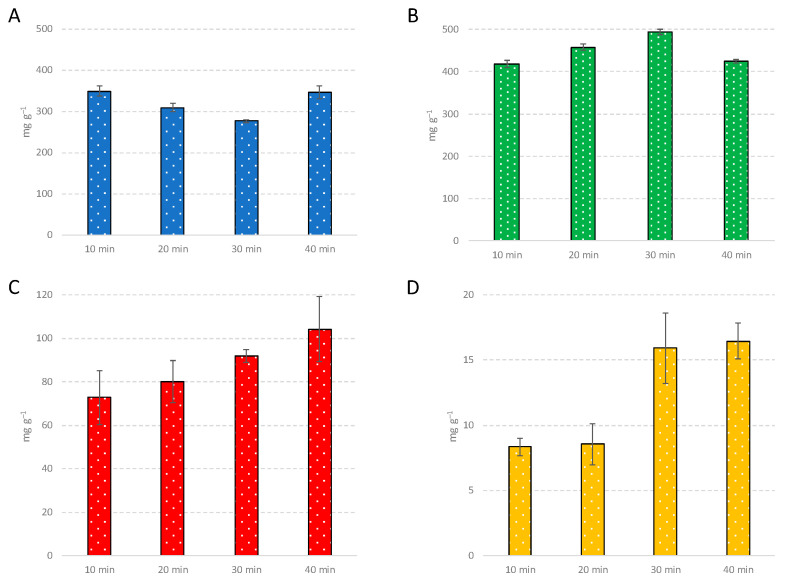
Level (mg g^−1^) of monoterpenes (**A**), sesquiterpenes (**B**), oxygenated compounds (**C**) and cannabinoids (**D**) obtained using different time periods of distillation at 700 W: 10 min, 20 min, 30 min and 40 min.

From the point of view of the chemical profile, the levels of monoterpenes, sesquiterpenes, oxygenated compounds, and cannabinoids were monitored at different time periods of distillation, as shown in Figure 1. Based on the obtained results, oxygenated compounds (especially sesquiterpenoids) and cannabinoids required longer distillation times for reaching the most abundant levels due to their higher molecular weights than terpenes. On the other hand, the faster approaches (10 min and 20 min) were revealed to be efficient in monoterpene and sesquiterpene cannabis EO enrichment. All single volatile components and chemical classes analyzed at different distillation programs are reported in Table S1.

On the basis of the observations reported so far, the microwave-based distillation method was optimized as follows: 1200 W for 10 min, and 700 W for 40 min. A clear and transparent oil distillate of light-yellow color, characterized by a good quality fragrance, was obtained. With respect to the traditional solvent-based extractions, the developed MADH method proved to be extremely advantageous not only in terms of practical aspects, but also in terms of cost and time-consumption. The operational simplicity combined to the lowest possible consumption of solvents and waste generation makes the method highly suitable for cannabis oil production.

### 2.2. Determination of Volatile Fraction in Cannabis EOs by Using GC-MS and GC-FID

Fresh and dried hemp inflorescences from different varieties, including Kompolti, Futura 75, Carmagnola, Felina 32, and Finola were extracted and isolated by using the previously optimized distillation program. The detailed compositions of each distilled oil were analyzed by using a gas chromatograph equipped with MS and FID detectors. Amounts of identified compounds, as well as chemical classes including hydrocarbons (monoterpenes, sesquiterpenes, diterpenes, and aliphatic) and oxygenated compounds (aldehydes, alcohols, esters, ketones, and cannabinoids), were investigated in detail (Table 1 and Table 2). The identity of components was revealed by using two different identification parameters. The first regarded the spectral MS similarity obtained when comparing the experimental spectra with those catalogued in commercial database, while the second parameter filtered the candidates on the correspondence of the LRIs listed in a dedicated library. This type of approach revealed, in an unequivocal manner, the identity of unknown compounds, ensuring an elevated degree of data reliability. In addition, the utilization of the double filter revealed the identity of compounds avoiding the utilization of authentic and pure standards for their confirmation. This concept assumes relevance both from the economic point of view and the unavailability of standards regularly detected in cannabis samples, e.g., selina-3,7-diene, α and γ-eudesmol, β and γ-selinene, and other terpenes [7]. A total of 165 compounds were identified, representing more than 90% of the entire volatile fraction. Absolute (mg g^−1^) and relative (FID area normalization) quantifications were carried out applying FID response factors (RFs) according to each chemical family. As reported in the literature, the FID system is defined as a “carbon counting device” [19] due to the fact that the response to hydrocarbons is proportional to the number of carbon atoms in the molecule. Variable responses are obtained for compounds containing heteroatoms. In fact, a carbon atom which is associated with a heteroatom (oxidized carbon) cannot produce a response to an FID detector; thus, this behavior is resolved by using a correction factor [20]. In such respect, FID peak areas of oxygenated compounds such as aldehydes, ketones and alcohols were corrected by a value of 1.3. In the case of epoxide and ester compounds, RFs of 1.5 and 1.6 were utilized, respectively. RF values were established on the base of previously consolidated procedures [21,22]. In the case of cannabinoids, for the first time, their contents have been determined with an FID detector by mean RFs. In such respect, cannabinoid standards were spiked with cannabigerorcin utilized as an internal standard (final concentration 100 mg L^−1^, except for Δ^9^-THC, 10 mg L^−1^) and injected consecutively for three runs. The formula used was:

$$RF=\frac{\left[analyte\right]}{\frac{{\mathrm{Area}}_{\;analyte}}{{\mathrm{Area}}_{\;ISTD}}\times \left[ISTD\right]\;}$$

where [*analyte*] represents the concentration of target cannabinoids (e.g., CBN or Δ^9^-THC), Area*_analyte_* is its absolute peak area, and [*ISTD*] and Area*_ISTD_* terms are the concentration and peak area of the cannabigerorcin internal standard, respectively. Experimental results demonstrated that the RFs of investigated cannabinoids, reported in Table 3, were counted as a value of 1.0. Absolute quantification was carried out by means of three different internal standards: nonane hydrocarbon (ISTD 1) was utilized to quantify the compounds eluted in the monoterpene region; nonadecane hydrocarbon (ISTD 2) for the quantification of components in the sesquiterpene region; and cannabigerorcin (ISTD 3) for determining the cannabinoid content in the last part of the GC chromatogram.

**Figure 2:**
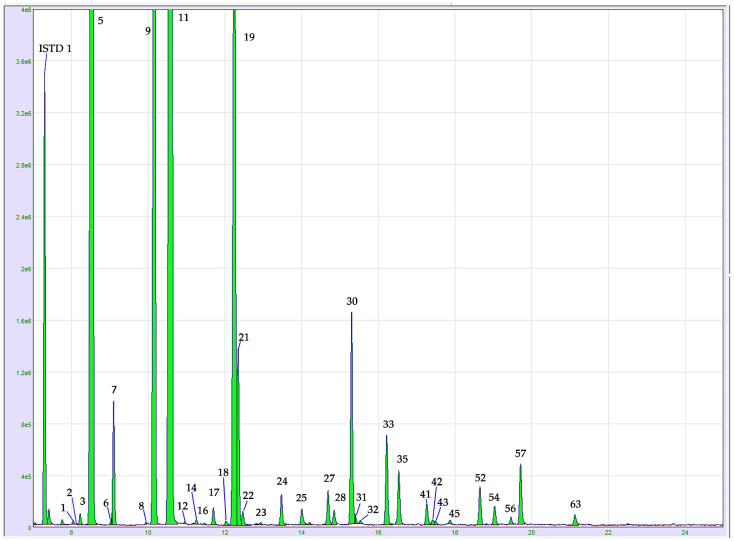
GC-MS chromatogram expansion of the monoterpene region in fresh hemp inflorescences, cv. Kompolti. For peak assignment, see the compounds listed in Table 1. Abbreviation: ISTD 1: nonane internal standard.

In accordance with data reported in the literature [10,18,23], hydrocarbons were the predominant chemical class with concentrations ranging from 563.45 ± 8.66 mg g^−1^ (64.35 ± 0.05%) registered in dried hemp Kompolti variety to 851.75 ± 59.81 mg g^−1^ (91.41 ± 0.03%) in fresh inflorescences of the Kompolti variety. Overall, fresh varieties revealed the higher monoterpene content, while their loss was detected in dried inflorescences, indicating that the drying processes caused the evaporation of compounds with low boiling points and altered the real composition of the starting material. An expansion of the GC-MS chromatogram relative to the monoterpene region is shown in Figure 2. The main components in term of abundance were α-pinene (peak 5), β-pinene (peak 9), myrcene (peak 11), and limonene (peak 19).

**Figure 3:**
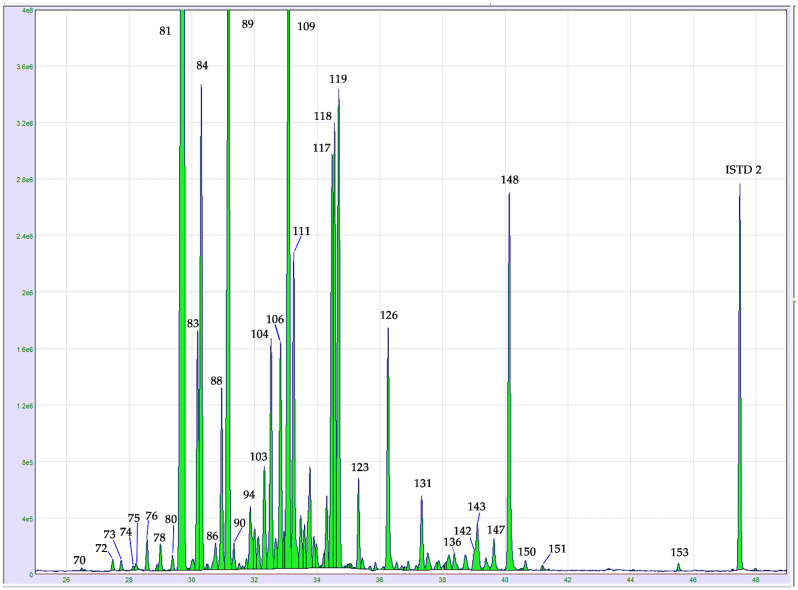
GC-MS chromatogram expansion of the sesquiterpenes region in dried hemp inflorescences, cv. Finola. For peak assignment, see the compounds listed in Table 1. Abbreviation: ISTD 2: nonadecane internal standard.

On the other hand, the sesquiterpene family was abundant in dried samples, reaching the maximum value of 676.94 ± 8.24 mg g^−1^ (71.53 ± 0.07%) in the Finola variety inflorescences (Figure 3). (*E*)-Caryophyllene (peak 81) and α-humulene (peak 89) were the predominant compounds. Relevant quantities of α-, (*E*)-bergamotene, (peak 83), α-guaiene (peak 84) and (*E*)-, β-farnesene (peak 88) were also registered. It is also worth noting the presence of selina and selinene derivatives as particularly abundant. In such respect, β-selinene (peak 104), α-selinene (peak 106), selina-4(15),7(11)-diene (peak 117) and selina-3,7(11)-diene (peak 119) were identified in distilled cannabis EOs. The detection of these compounds was also reported by Benelli et al. [23] after the distillation of *Cannabis Sativa* cv. Felina 32. Additionally, Marchinini et al. [24] detected abundant quantities of these derivatives both in herb inflorescences and hashish samples using a comprehensive two-dimensional GC system coupled to mass spectrometer (GC × GC-MS).

A high variability was involved in the oxygenated compounds with concentrations ranging from 65.86 ± 1.11 mg g^−1^ (4.69 ± 0.13%) in fresh hemp variety Futura 75, to 321.78 ± 4.39 mg g^−1^ (26.66 ± 0.06%) in the dried Kompolti variety. Generally, alcohols and epoxides were the most abundant compounds. The former group was abundant, especially in fresh hemp cultivar Carmagnola, including linalool (peak 30 in Figure 2) as the main monoterpene alcohol, while guaiol, γ-eudesmol, β-eudesmol, bulnesol and α-bisabolol (peak 148 in Figure 3) were the predominant sesquiterpene alcohols. Among the alcohols, it is also worth highlighting the presence of (*E*)-nerolidol (peak 123 in Figure 3) whose content was abundant, especially in dried hemp cultivar Kompolti (33.68 ± 0.59 mg g^−1^). On the other hand, the epoxide family registered high levels especially in dried hemp inflorescences, according to the oxidation processes caused by drying treatments. Cariophyllene oxide (peak 126) and humulene epoxide (peak 131) were the main epoxides identified in analyzed varieties. In the case of the hemp Felina 32 variety, caryophyllene oxide reached a value as much as of 88.75 ± 2.03 mg g^−1^ (6.62 ± 0.00%), while lower levels were detected for humulene epoxide (27.35 ± 0.58 mg g^−1^ corresponding to 2.04 ± 0.00%).

**Figure 4:**
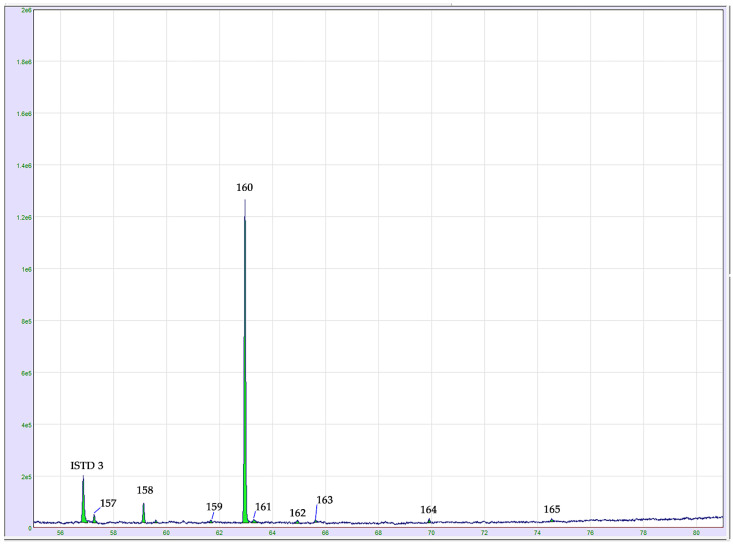
GC-MS chromatogram expansion of the cannabinoid region in dried hemp inflorescences, cv. Kompolti. For peak assignment, see the compounds listed in Table 1. Abbreviation: ISTD 3: cannabigerorcin internal standard.

Microwave-based distillation also allowed the isolation of cannabinoid compounds. In fact, a total of seven cannabinoids were identified in selected cannabis EOs. For a detailed elucidation of cannabinoids, pure standards of cannabidivarin (CBDV), cannabicitran (CBT), cannabicyclol (CBL), cannabidiol (CBD), cannabichromene (CBC), δ8-tetrahydrocannabinol (Δ^8^-THC) and δ9-tetrahydrocannabinol (Δ^9^-THC) were injected into the GC instrument and their MS spectra and reference LRI values were determined. From the quantitative point of view, cannabidiol (CBD, peak 160 in Figure 4) was the predominant cannabinoid, in accordance with the non-drug-type nature of analyzed samples. On the base of this result, the technique demonstrated to also be adept to distinguish *Cannabis sativa* varieties cultivated for fiber production (hemp) or medical and drug purposes (marijuana). Overall, a low content of cannabinoids was quantified in fresh samples (from 2.11 ± 0.06 mg g^−1^ to 5.33 ± 0.28 mg g^−1^), while higher levels were obtained in those dried (from 12.74 ± 1.73 mg g^−1^ to 22.95 ± 1.17 mg g^−1^). This behavior may be related to the higher content of water in fresh material which produces the lowest yields of cannabinoids due to their poor solubility [25]. In addition, no trace of cannabinoids in acid form was detected in analyzed samples. According to their thermal lability, the high temperature to which the molecules are exposed during drying processes, distillation (including MAHD), injection, and chromatographic runs causes the decarboxylation of the native acid cannabinoids into their neutral form [18]. δ^9^-THC (peak 163 in Figure 4), responsible for the psychoactive effects of the cannabis plant [26], was found in dried hemp inflorescences in quantities ranging from 0.18 ± 0.00 mg g^−1^ to 0.56 ± 0.08 mg g^−1^. An expansion of the GC-MS chromatogram of the cannabinoid region is illustrated in Figure 4.

### 2.3. Enantiomeric Distribution in Cannabis EO

The chirality concept assumes relevance in control laboratories if the enantiomeric distributions of target compounds are well-defined. In this case, it became possible establish the authenticity of suspicious samples. The enantio-GC strategy developed here can be considered the key to reveal eventual adulteration in cannabis EO. As frequently reported in the literature, the chiral investigations of terpenes and terpenoids are carried out by using cyclodextrin-based stationary phases, capable of separating both dextrorotary (+) and levorotary (−) forms of the enantiomers [15]. In order to guarantee the reliability of the analytical data and provide a highly valid chromatographic approach, commercial standards of known absolute configuration were initially injected in GC-MS. When not available, aromatic plant EOs, such as bergamot and cabreuva EOs, were analyzed for establishing the elution order of the individual enantiomers. To this purpose, a 30 m capillary column coated with a permethylated β-cyclodextrin stationary phase was used and a dedicated chiral MS database was constructed in the laboratory. In order to correctly characterize enantiomeric distribution, avoiding mistaken peak assignment, LRIs of the separated chiral species were calculated against a homologue series of C_8_–C_20_ saturated alkanes.

**Figure 5:**
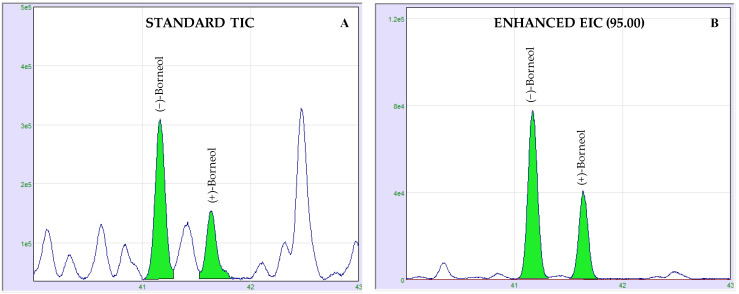
Enantio-GC comparison of zoomed borneol region of standard total ion current (TIC) (**A**) and enhanced extracted ion current (EIC) (**B**) in cannabis EO. Borneol enantiomer ratio was determined monitoring the 95 *m*/*z* fragment (**B**).

A total of 10 enantiomer couples were determined in cannabis EOs, and their relative abundances are reported in Table 4. The enantiomeric excesses of α-pinene, camphene, β-pinene, limonene, linalool and fenchyl alcohol were calculated monitoring their total ion current (TIC). In the case of borneol, (*E*)-caryophyllene, (*E*)-nerolidol and caryophyllene oxide, the TIC signals were characterized from numerous interferences and/or coelution caused by the high complexity of the analyzed sample, especially in the sesquiterpene region. Thus, the authors preferred to select only one *m/z* value, usually corresponding to the most abundant fragment, and create an extracted ion current (EIC) chromatogram to facilitate the data processing. In fact, EIC chromatograms improved the visualization of the signal produced, allowing the correct evaluation of both (+) and (−) forms. Applications of the enhanced EIC chromatogram are illustrated in Figure 5.

In accordance with the major parts of the plants, *Cannabis sativa* species showed a highly typical trend. The first part of enantio-GC chromatogram was characterized by the presence of monoterpenes including α-pinene, camphene, β-pinene and limonene. In elution order, α-pinene showed an excess of the dextrorotary form among cultivars, except for the hemp variety Carmagnola. The abundance of the (+) form was counted from a maximum value of 95.97% in Kompolti fresh inflorescences to a minimum value of 87.89% in the Finola variety. An inversion of the enantiomer distribution was observed in the case of the Carmagnola variety, with a value of the (−) isomer of 94.02%. A regular trend was registered in the case of camphene enantiomers. In such respect, a clear predominance of (+) or (−) forms was not found, and their abundance varied around 50%. However, a high enantiomer excess was registered in favor of (−)-camphene in hemp variety Carmagnola, indicating an irregular behavior compared to other cannabis samples. The values determined for the enantiomeric excess of β-pinene and limonene were constant between the investigated cultivars. In detail, the former component presented an abundance of the dextrorotary isomer with values ranging from 68.07% in Finola to 97.34% in Carmagnola, while the second compound was abundant in the levorotary form (from 73.22% in dried hemp variety Futura 75 to 94.02% in Carmagnola). In the intermediate region of the enantio-GC chromatogram, a group of alcohol compounds, including linalool, fenchyl alcohol and borneol, was investigated. The (+) form of linalool and fenchyl alcohol were abundant in distilled samples, showing a linear behavior inter-cultivar. Regarding borneol, the enantiomeric excess of the (−) form indicated a clear prevalence of levorotary isomer in cannabis plants. (*E*)-Caryophyllene, (*E*)-nerolidol, and caryophyllene oxide were eluted in the last part of the chromatogram grouped in the sesquiterpene region. Due to the absence of the (+) form of (*E*)-caryophyllene, not only as commercial standard but also as a compound rarely available in nature [27], its enantiomeric excess was tentatively determined. The exclusive commercially available (−)-(*E*)-caryophyllene standard allowed us to obtain information about its elution time, but no values regarding the resolving power of the chiral column were obtainable. From the obtained results, (*E*)-caryophyllene showed an enantiomeric excess of 100% in favor of the (−) form and no trace of the (+) isomer was detected in the enantio-GC-MS chromatogram. An identical result was obtained by Fiorini et al. [13]. For evaluating the enantiomeric distribution of (*E*)-nerolidol in *Cannabis Sativa* plants, an (*E*)-nerolidol racemic mixture and a *Cabreuva* (*Myrocarpus fastigiatus*) oil containing mainly (+)-(*E*)-nerolidol [28] were utilized. Enantio-GC analysis of the standard showed the capability of the column to separate both (−) and (+) forms of (*E*)-nerolidol, while *Cabreuva* EO analysis allowed us to establish the elution order of the enantiomers. The chiral results showed an excess of (+)-(*E*)-nerolidol with abundances ranging from 95.02% in fresh hemp cultivar Kompolti to 99.44% in dried Kompolti. The chiral separation of caryophyllene oxide was confirmed by the injection of the certified standard (−)-caryophyllene oxide, containing trace levels of (+) form. According to data in the literature [13], the enantio-GC-MS method confirmed the predominance of (−)-caryophyllene oxide in *Cannabis sativa* plant. Low levels of (+)-caryophyllene oxide (min value, 0.98%–max value, 8.57%) were reported for the first time.

## 3. Materials and Methods

### 3.1. Chemicals and Reagents

Dry and fresh hemp inflorescences of different cultivars registered in the EU Plant Variety Database [29], including 2 monoecious (Futura 75 and Felina 32) and 3 dioecious (Kompolti, Carmagnola and Finola) varieties were provided by Canapar Group, Ragusa, Italy. A genuine cold-pressed bergamot EO, used for determining the elution order of enantiomers of α-pinene, camphene and linalool, was kindly supplied by Simone Gatto s.r.l. (Messina, Italy). Cabreuva (*Myrocarpus fastigiatus*) EO, utilized for establishing the elution order of (+) and (−)-(*E*)-nerolidol, was purchased from Berjè (Carteret, NJ, USA). *n*-Heptane (for HPLC, ≥99%) was purchased from Merck Life Science (Merck KGaA, Darmstadt, Germany). Ultrapure water was obtained from Milli-Q advantage A10 system (Millipore, Bedford, MA, USA). Pure standards of cannabidivarin (CBDV), cannabidiol (CBD), δ8-tetrahydrocannabinol (Δ^8^-THC), cannabichromene (CBC) and δ9-tetrahydrocannabinol (Δ^9^-THC) were purchased from Merck Life Science, while cannabicyclol (CBL) and cannabicitran (CBT) standards were acquired from Cayman Chemical (Ann Arbor, MI, USA). Nonane (ISTD 1) and nonadecane (ISTD 2) hydrocarbons were purchased from Merck Life Science, while cannabigerorcin (ISTD 3) was from Cayman Chemical. Terpene and terpenoids standards of (1*S*)-((−))-β-pinene (≥97%, FCC, FG), (*R*)-(+)-limonene (97%), (1*R*)-(+)-fenchol (analytical standard), (1S)-(−)-borneol, (−)-(*E*)-caryophyllene (≥80%, FCC, FG) and (−)-caryophyllene oxide (95%) was purchased from Merck Life Science. (*E*)-Nerolidol (≥90%) standards was purchased from Extrasynthese (Genay, France). C_7_–C_40_ and C_8_–C_20_ saturated alkane standards (Merck Life Science) were used for the LRI calculations on SLB-5ms and β-Dex 120 chiral columns, respectively.

### 3.2. Distillation System

Masses of 400 g of hemp inflorescences were rehydrated using 1.2 L of ultrapure water (ratio 1:3 sample:water). After 30 min of mixing and soaking, the entire biomass was transferred in a 5 L ETHOS-X glass reactor and closed using a glass cover with an intermediate silicone o-ring and a PTFE) sealing kit. Afterwards, the reactor was placed into the Milestone “Ethos X” instrument (Milestone, Sorisole, Italy), equipped with two magnetrons capable of developing a maximum power of 1800 W and an infrared sensor for monitoring the internal heating. A Clevenger-type apparatus was connected outside the microwave instrument and allowed the condensation of the distillated oil through a circulating water system maintained at a temperature of 8 °C through a chiller. The cooling system allowed not only the EO isolation, but also a continuous reflux of the evaporated water to the reactor, restoring the water content to the hemp material. The distillation program was optimized as follows: 10 min at 1200 W and 40 min at 700 W. Finally, the distilled cannabis EO was collected from the distillation module by using a Pasteur pipette and transferred in glass autosampler vial. In order to favor the water–oil separation, the distillate was frozen.

### 3.3. Sample Preparation

A certified analytical balance (AX204 Mettler Toledo, d = 0.1 mg) was used to prepare internal standard solutions at known concentrations. In detail, 10 mg of pure standard nonane (ISTD 1) and nonadecane (ISTD 2) were weighed and solubilized in a 10 mL volumetric flask using *n*-heptane. Cannabigerorcin (ISTD 3) was solubilized in ethanol at a concentration of 1000 mg L^−1^.

For compound quantification (mg g^−1^), 50 μL of cannabigerorcin solution were inserted in an autosampler vial using a high-precision Hamilton syringe (volume 50 μL). The ethanolic solvent was turned away using a constant nitrogen flow. Then, 200 μL of nonane and nonadecane heptanic solution were collected and dispensed into the vial using a Hamilton syringe (volume 250 μL); subsequently, 10 mg of distilled cannabis EO was added to the vial and solubilized in 800 μL (1:100 dilution) *n*-heptane. The sample was homogenized using a vortex mixer and injected into the GC system. For a major data precision, each cannabis EO was prepared in three replicates.

### 3.4. GC-MS/FID Analysis

The separation, identification, and quantification of terpenes, terpenoids and cannabinoids in distilled cannabis EOs was carried out by using a Clarus 680 GC (PerkinElmer Inc., Waltham, MA, USA) coupled to Clarus SQ 8T single quad mass spectrometer and FID detector. For chromatographic separation, the GC system was equipped a low polarity capillary column, named SLB-5ms 30 m × 0.25 mm *id* × 0.25 µm *df* (Merck Life Science). A “Y” splitting unit was connected at the column outlet and it allowed to the eluate to be simultaneously split in the MS (40% of the total flow) and FID (60% of the total flow) detectors. Specifically, two uncoated segments were connected to the Y splitting unit: 1 m × 0.1 mm *id*, and 1.85 m × 0.1 mm *id* fused silica capillaries to MS and FID, respectively. The injector temperature was set at 280 °C. The temperature program was as follows: 50 °C to 350 °C at 3.0 °C min^−1^. Injection volume was of 1.0 µL with a split ratio of 1:10. Helium was used as a gas carrier at a constant linear velocity of 30 cm s^−1^ and an inlet pressure of 224 kPa. MS parameters were as follows: mass range 40–550 amu, source temperature: 220 °C, GC interface temperature: 250 °C, scan time: 0.2 sec. The FID temperature was set at 300 °C (sampling rate: 200 ms). FID detector gas flows were as follows: 45 mL min^−1^ for H_2_ and 450 mL min^−1^ for air. TurboMass software (version 6.1.2.2048, PerkinElmer) was used for data acquisition, while FID and MS data processing was carried out by using ChromatoPlus Spectra (version 8.1.3, Dani Analitica, Milan, Italy) from a previous conversion of a data file in cdf format. A homologous series C_7_–C_40_ saturated alkane standard mixture (Merck Life Science) in hexane (1000 g mL^−1^) was used for LRI calculations on the SLB-5ms column, useful for the identification of analytes. In this regard, the peak assignment was carried out through the utilization of two different identification parameters: reverse match (over 850) and LRI filter (±5). The FFNSC 4.0 mass spectral database (Chromaleont, Messina, Italy) was mainly used for the identification of terpene and terpenoid components, while the identity of cannabinoids was investigated by using a lab-constructed mass spectral library.

### 3.5. Enantio-GC-MS Analysis

Enantiomeric distribution of cannabis EOs was investigated by using a Clarus 680 GC (PerkinElmer Inc.) couples to a Clarus SQ 8T single quad mass spectrometer. The chiral separation was carried out using a cyclodextrin-based capillary column, named beta-DEX™ 120, 30 m × 0.25 mm *id* × 0.25 μm *df* (Merck Life Science). The temperature program was as follows: 50 °C to 220 °C at 2.0 °C min^−1^. The injection volume was of 1.0 µL with a split ratio of 1:10, and the injector temperature was 220 °C. Helium was used as the gas carrier at a constant linear velocity of 30 cm s^−1^ and an inlet pressure of 26.7 kPa. MS parameters were as follows: mass range 40–550 amu, source temperature: 220 °C, GC interface temperature: 250 °C, scan time: 0.2 s. TurboMass software (version 6.1.2.2048, PerkinElmer) was used for data collection, while MS data handling was carried out by using ChromatoPlus Spectra (version 8.1.3, Dani Analitica, Milan, Italy). A homologous series of C_8_–C_20_ alkane standard solution (Merck Life Science) in hexane (~40 mg L^−1^ each) was used for the LRI calculations of dextrorotary and laevorotary enantiomers. In a similar manner to that previously reported, the identification of the enantiomeric couples was performed using spectra reverse match (over 850) and LRI filter (±3) filters. A chiral lab-constructed mass spectral database including enantio-LRIs was used for identifying chiral compounds.

## 4. Conclusions

The present research has demonstrated that MAHD is an effective technique for the extraction and isolation of cannabis EO from fresh and dried hemp inflorescences. The developed protocol can represent a reliable and profitable method for those industries interested in the flavor and fragrance market of cannabis EO. Remarkable advantages in terms of operational simplicity, cost, and time-efficiency characterized the distillation procedure. At the same time, it is worth empathizing the ecological aspects of the methodology according to green chemistry principles such as minimizing toxicity, waste production and saving energy. In addition, the authors have reported a detailed study about the GC-MS/FID analysis adapted for establishing the fingerprint of terpene, terpenoid and cannabinoid compounds. Absolute quantification of single compounds was performed by using the internal standard method applying FID response factors in accordance with each chemical family, including those of cannabinoid. In order to reveal an eventual adulteration or human interferences, an enantio-GC method was optimized and the enantiomeric distribution of 10 chiral couples were well-defined.

## Figures and Tables

**Table 1:** List of volatile compounds detected in cannabis essential oils (EOs) distilled from fresh and dried inflorescences of Kompolti and Futura 75 cultivars. Abbreviations: R.F.: response factor; match: database spectral matching; LRI_exp_: experimental LRI; LRI_ref_: reference LRI. Quantitative results are expressed both in absolute manner (mg g^−1^) and in relative percentages (%). The compounds are also grouped in monoterpene, sesquiterpene, diterpene and aliphatic hydrocarbons and as well as oxygenated compounds including aldehyde, alcohol, ester, ketone, epoxide, and cannabinoid. The cannabis EO yields are also reported.

ID	Name	Chemical Subclass	R.F.	Match	LRI_exp_	LRI_ref_	Fresh Kompolti		Dried Kompolti		Fresh Futura 75		Dried Futura 75	
							mg g^−1^	%	mg g^−1^	%	mg g^−1^	%	mg g^−1^	%
1	Hashishene	Monoterpene	1.0	947	921	921	0.19 ± 0.02	0.02 ± 0.00	1.35 ± 0.01	0.15 ± 0.00	2.78 ± 0.01	0.30 ± 0.00	0.71 ± 0.02	0.08 ± 0.00
2	Tricyclene	Monoterpene	1.0	931	924	923	0.06 ± 0.01	0.01 ± 0.00	-	-	-	-	-	-
3	α-Thujene	Monoterpene	1.0	991	927	927	0.40 ± 0.03	0.04 ± 0.00	-	-	0.53 ± 0.01	0.06 ± 0.00	0.39 ± 0.01	0.04 ± 0.00
4	Thuja-2,4(10)-diene	Monoterpene	1.0	950	954	953	-	-	-	-	-	-	-	-
5	α-Pinene	Monoterpene	1.0	980	935	933	270.14 ± 23.12	29.33 ± 0.04	21.43 ± 0.05	2.37 ± 0.02	181.89 ± 0.64	19.50 ± 0.32	117.81 ± 2.65	12.86 ± 0.08
6	α-Fenchene	Monoterpene	1.0	976	951	950	0.13 ± 0.01	0.01 ± 0.00	0.08 ± 0.01	0.01 ± 0.00	0.20 ± 0.01	0.02 ± 0.00	0.16 ± 0.00	0.02 ± 0.00
7	Camphene	Monoterpene	1.0	995	951	953	4.50 ± 0.38	0.49 ± 0.00	0.88 ± 0.01	0.10 ± 0.00	3.35 ± 0.01	0.36 ± 0.01	2.06 ± 0.04	0.22 ± 0.00
8	Sabinene	Monoterpene	1.0	886	974	972	0.20 ± 0.02	0.02 ± 0.00	-	-	0.72 ± 0.00	0.08 ± 0.00	0.24 ± 0.01	0.03 ± 0.00
9	β-Pinene	Monoterpene	1.0	986	980	978	104.13 ± 8.90	11.31 ± 0.01	5.71 ± 0.04	0.63 ± 0.00	54.17 ± 0.12	5.81 ± 0.09	22.54 ± 0.48	2.46 ± 0.01
10	6-methyl-Hept-5-en-2-one	Ketone	1.3	920	986	986	-	-	0.33 ± 0.01	0.03 ± 0.00	-	-	0.24 ± 0.01	0.02 ± 0.00
11	Myrcene	Monoterpene	1.0	982	993	991	300.38 ± 25.44	32.62 ± 0.02	10.83 ± 0.10	1.20 ± 0.00	143.12 ± 0.37	15.34 ± 0.23	61.81 ± 1.24	6.75 ± 0.03
12	*m*-Mentha-1(7), 8-diene	Monoterpene	1.0	873	1002	1001	0.11 ± 0.01	0.01 ± 0.00	-	-	-	-	-	-
13	*n*-Octanal	Aldehyde	1.3	975	1006	1006	-	-	-	-	-	-	-	-
14	α-Phellandrene	Monoterpene	1.0	978	1008	1007	0.20 ± 0.02	0.02 ± 0.00	0.30 ± 0.01	0.03 ± 0.00	2.13 ± 0.02	0.23 ± 0.01	0.55 ± 0.01	0.06 ± 0.00
15	δ3-Carene	Monoterpene	1.0	986	1011	1009	-	-	0.83 ± 0.01	0.09 ± 0.00	6.02 ± 0.01	0.65 ± 0.01	27.03 ± 0.54	2.95 ± 0.01
16	hexyl-Acetate	Ester	1.6	981	1013	1012	0.16 ± 0.01	0.01 ± 0.00	-	-	-	-	-	-
17	α-Terpinene	Monoterpene	1.0	983	1019	1018	0.60 ± 0.08	0.07 ± 0.00	0.30 ± 0.01	0.03 ± 0.00	1.71 ± 0.01	0.18 ± 0.00	0.63 ± 0.01	0.07 ± 0.00
18	*p*-Cymene	Monoterpene	1.0	963	1026	1025	0.16 ± 0.02	0.02 ± 0.00	0.72 ± 0.01	0.08 ± 0.00	0.94 ± 0.00	0.10 ± 0.00	2.78 ± 0.05	0.30 ± 0.00
19	Limonene	Monoterpene	1.0	991	1031	1030	74.26 ± 6.26	8.06 ± 0.00	6.53 ± 0.31	0.71 ± 0.00	21.51 ± 0.05	2.31 ± 0.03	11.23 ± 0.22	1.23 ± 0.00
20	β-Phellandrene	Monoterpene	1.0	975	1032	1031			0.54 ± 0.03	0.06 ± 0.00	5.87 ± 0.03	0.63 ± 0.01	-	-
21	Eucalyptol	Alcohol	1.3	984	1034	1032	7.29 ± 0.68	0.61 ± 0.01	3.06 ± 0.04	0.26 ± 0.00	-	-	1.94 ± 0.04	0.16 ± 0.00
22	(*Z*)-, β-Ocimene	Monoterpene	1.0	951	1036	1035	0.10 ± 0.01	0.01 ± 0.00	0.08 ± 0.01	0.01 ± 0.00	4.72 ± 0.01	0.51 ± 0.01	7.75 ± 0.16	0.85 ± 0.00
23	(*E*)-, β-Ocimene	Monoterpene	1.0	980	1047	1046	0.02 ± 0.00	0.00 ± 0.00	2.08 ± 0.03	0.23 ± 0.00	33.59 ± 0.07	3.60 ± 0.04	23.05 ± 0.43	2.52 ± 0.01
24	γ-Terpinene	Monoterpene	1.0	985	1060	1058	1.22 ± 0.10	0.13 ± 0.00	0.48 ± 0.01	0.05 ± 0.00	1.67 ± 0.01	0.18 ± 0.00	0.81 ± 0.02	0.09 ± 0.00
25	(*Z*)-Sabinene hydrate	Alcohol	1.3	963	1072	1069	1.03 ± 0.08	0.09 ± 0.00	0.89 ± 0.01	0.08 ± 0.00	1.00 ± 0.01	0.08 ± 0.00	1.06 ± 0.01	0.09 ± 0.00
26	Octanol	Alcohol	1.3	920	1072	1076	-	-	-	-	-	-	-	-
27	Terpinolene	Monoterpene	1.0	992	1088	1086	1.45 ± 0.12	0.16 ± 0.00	1.22 ± 0.02	0.14 ± 0.00	58.46 ± 0.19	6.27 ± 0.07	4.74 ± 0.09	0.52 ± 0.00
28	Fenchone	Ketone	1.3	990	1091	1090	0.90 ± 0.08	0.08 ± 0.00	0.18 ± 0.05	0.02 ± 0.00	-	-	-	-
29	*p*-Cymenene	Monoterpene	1.0	964	1093	1093	-	-	0.53 ± 0.03	0.06 ± 0.00	0.68 ± 0.01	0.07 ± 0.00	0.13 ± 0.01	0.01 ± 0.00
30	Linalool	Alcohol	1.3	994	1102	1101	12.17 ± 0.99	1.02 ± 0.00	2.86 ± 0.04	0.24 ± 0.00	1.83 ± 0.01	0.15 ± 0.00	2.42 ± 0.04	0.20 ± 0.00
31	(*E*)-Sabinene hydrate	Alcohol	1.3	976	1104	1099	0.38 ± 0.05	0.03 ± 0.00	0.39 ± 0.05	0.03 ± 0.00	-	-	0.42 ± 0.02	0.03 ± 0.00
32	*n*-Nonanal	Aldehyde	1.3	972	1107	1107	0.29 ± 0.04	0.02 ± 0.00	1.46 ± 0.03	0.12 ± 0.00	0.50 ± 0.01	0.04 ± 0.00	0.62 ± 0.02	0.05 ± 0.00
33	Fenchyl alcohol	Alcohol	1.3	996	1122	1123	4.61 ± 0.37	0.39 ± 0.00	4.09 ± 0.09	0.35 ± 0.01	1.21 ± 0.02	0.10 ± 0.00	1.22 ± 0.02	0.10 ± 0.00
34	(*E*)-, *p*-Mentha-2,8-dien-1-ol	Alcohol	1.3	954	1125	1122	-	-	-	-	0.18 ± 0.00	0.02 ± 0.00	-	-
35	(*Z*)-, *p*-Menth-2-en-1-ol	Alcohol	1.3	915	1129	1124	2.95 ± 0.24	0.25 ± 0.00	2.57 ± 0.03	0.22 ± 0.00	-	-	-	-
36	*allo*-Ocim-(4E,6Z)-ene	Monoterpene	1.0	977	1130	1128	-	-	-	-	1.48 ± 0.02	0.16 ± 0.00	1.40 ± 0.03	0.15 ± 0.00
37	Limona ketone	Ketone	1.3	967	1128	1131	-	-	-	-	-	-	-	-
38	(*Z*)-, *p*-Mentha-2,8-dien-1-ol	Alcohol	1.3	980	1139	1138	-	-	1.86 ± 0.02	0.16 ± 0.00	-	-	0.45 ± 0.08	0.03 ± 0.00
39	(*E*)-Myroxide	Epoxide	1.5	973	1141	1141	-	-	-	-	-	-	0.52 ± 0.05	0.04 ± 0.00
40	(*E*)-Pinocarveol	Alcohol	1.3	992	1144	1141	-	-	3.50 ± 0.21	0.30 ± 0.02	-	-	0.13 ± 0.01	0.01 ± 0.00
41	Ipsdienol	Alcohol	1.3	985	1145	1146	1.63 ± 0.13	0.14 ± 0.00	-	-	-	-	-	-
42	Myrcenone	Ketone	1.3	924	1148	1149	0.62 ± 0.05	0.05 ± 0.00	-	-	0.11 ± 0.01	0.01 ± 0.00	-	-
43	*neo*-Isopulegol	Alcohol	1.3	914	1150	1148	0.21 ± 0.02	0.02 ± 0.00	-	-	-	-	-	-
44	(*Z*)-Pinene hydrate	Alcohol	1.3	943	1149	1144	-	-	-	-	-	-	-	-
45	Camphene hydrate	Alcohol	1.3	977	1158	1156	0.21 ± 0.02	0.02 ± 0.00	-	-	-	-	-	-
46	(*E*)-Verbenol	Alcohol	1.3	940	1149	1145	-	-	0.31 ± 0.01	0.03 ± 0.00	0.29 ± 0.03	0.02 ± 0.00	0.16 ± 0.01	0.01 ± 0.00
47	β-Pinene oxide	Epoxide	1.5	926	1153	1156	-	-	0.64 ± 0.01	0.05 ± 0.00	0.74 ± 0.01	0.05 ± 0.00	0.18 ± 0.01	0.01 ± 0.00
48	Camphene hydrate	Alcohol	1.3	910	1158	1156	-	-	0.42 ± 0.01	0.04 ± 0.00	-	-	-	-
49	Menthone	ketone	1.3	981	1159	1158	-	-	-	-	-	-	1.55 ± 0.03	0.13 ± 0.00
50	(*E*)-Pinocamphone	ketone	1.3	934	1163	1160	-	-	0.25 ± 0.01	0.02 ± 0.00	-	-	-	-
51	Pinocarvone	ketone	1.3	981	1166	1164	-	-	1.09 ± 0.02	0.09 ± 0.00	0.26 ± 0.01	0.02 ± 0.00	-	-
52	Borneol	Alcohol	1.3	998	1175	1173	2.14 ± 0.17	0.18 ± 0.00	2.66 ± 0.03	0.23 ± 0.00	0.92 ± 0.01	0.08 ± 0.00	-	-
53	Menthol	Alcohol	1.3	986	1180	1184	-	-	-	-	-	-	1.56 ± 0.06	0.13 ± 0.00
54	Terpinen-4-ol	Alcohol	1.3	982	1183	1184	1.09 ± 0.10	0.09 ± 0.00	0.99 ± 0.02	0.08 ± 0.00	0.85 ± 0.01	0.07 ± 0.00	0.85 ± 0.07	0.07 ± 0.01
55	*p*-Cymen-8-ol	Alcohol	1.3	913	1190	1189	-	-	0.95 ± 0.01	0.08 ± 0.00	0.85 ± 0.01	0.07 ± 0.00	1.18 ± 0.07	0.10 ± 0.01
56	hexyl-Butyrate	Ester	1.6	988	1193	1195	0.53 ± 0.05	0.04 ± 0.00	-	-	1.20 ± 0.03	0.08 ± 0.00	0.52 ± 0.07	0.04 ± 0.01
57	α-Terpineol	Alcohol	1.3	993	1198	1195	3.50 ± 0.28	0.29 ± 0.00	2.46 ± 0.02	0.21 ± 0.00	0.93 ± 0.01	0.08 ± 0.00	1.02 ± 0.06	0.09 ± 0.01
58	Myrtenol	Alcohol	1.3	860	1200	1202	-	-	0.95 ± 0.02	0.08 ± 0.00	-	-	-	-
59	octyl-Acetate	Ester	1.6	970	1212	1214	-	-	-	-	-	-	-	-
60	(*E*)-Piperitol	Alcohol	1.3	936	1213	1209	-	-	0.34 ± 0.04	0.03 ± 0.00	-	-	-	-
61	*endo*-Fenchyl acetate	Ester	1.6	897	1220	1221	-	-	-	-	-	-	-	-
62	(*E*)-Carveol	Alcohol	1.3	934	1223	1223	-	-	0.56 ± 0.01	0.05 ± 0.00	-	-	-	-
63	Citronellol	Alcohol	1.3	992	1229	1232	0.89 ± 0.07	0.07 ± 0.00	0.61 ± 0.01	0.05 ± 0.00	0.72 ± 0.01	0.06 ± 0.00	0.63 ± 0.03	0.05 ± 0.00
64	Dec-(4*Z*)-en-1-ol	Alcohol	1.3	976	1256	1258	0.18 ± 0.02	0.02 ± 0.00	-	-	-	-	-	-
65	Dec-(4*E*)-en-1-ol	Alcohol	1.3	994	1259	1262	-	-	-	-	-	-	-	-
66	Decyl alcohol	Alcohol	1.3	952	1275	1278	-	-	-	-	-	-	-	-
67	Bornyl Acetate	Ester	1.6	975	1286	1285	-	-	0.45 ± 0.01	0.03 ± 0.00	-	-	-	-
68	Cogeijerene	Sesquiterpene	1.0	920	1288	1286	-	-	-	-	-	-	-	-
69	*n*-Tridecane	Aliphatic	1.0	950	1301	1300	-	-	-	-	-	-	-	-
70	α-Cubebene	Sesquiterpene	1.0	968	1350	1347	-	-	-	-	-	-	-	-
71	Eugenol	Alcohol	1.3	933	1356	1357	-	-	0.55 ± 0.01	0.04 ± 0.00	-	-	-	-
72	α-Ylangene	Sesquiterpene	1.0	964	1372	1371	-	-	2.94 ± 0.05	0.34 ± 0.00	0.93 ± 0.07	0.10 ± 0.00	1.82 ± 0.01	0.20 ± 0.00
73	α-Copaene	Sesquiterpene	1.0	956	1372	1375	0.12 ± 0.01	0.01 ± 0.00	0.62 ± 0.02	0.07 ± 0.00	0.47 ± 0.06	0.05 ± 0.00	0.39 ± 0.01	0.04 ± 0.00
74	hexyl-Hexanoate	Ester	1.6	985	1387	1390	-	-	-	-	1.62 ± 0.11	0.17 ± 0.00	0.22 ± 0.02	0.02 ± 0.00
75	7-*epi*-Sesquithujene	Sesquiterpene	1.0	942	1389	1387	-	-	-	-	0.79 ± 0.08	0.08 ± 0.00	0.15 ± 0.03	0.02 ± 0.00
76	Sativene	Sesquiterpene	1.0	905	1397	1394	0.30 ± 0.02	0.03 ± 0.00	0.80 ± 0.02	0.09 ± 0.00	0.35 ± 0.05	0.04 ± 0.00	0.62 ± 0.01	0.07 ± 0.00
77	α-Funebrene	Sesquiterpene	1.0	909	1404	1403	-	-	0.28 ± 0.01	0.03 ± 0.00	0.35 ± 0.04	0.04 ± 0.00	0.36 ± 0.01	0.04 ± 0.00
78	(*Z*)-Caryophyllene	Sesquiterpene	1.0	953	1407	1413	-	-	5.13 ± 0.09	0.59 ± 0.00	2.31 ± 0.13	0.24 ± 0.00	2.78 ± 0.01	0.30 ± 0.00
79	α-Gurjunene	Sesquiterpene	1.0	986	1410	1406	-	-	0.58 ± 0.01	0.07 ± 0.00	0.51 ± 0.04	0.05 ± 0.00	0.34 ± 0.01	0.04 ± 0.00
80	α-, (*Z*)-Bergamotene	Sesquiterpene	1.0	978	1415	1416	0.20 ± 0.01	0.02 ± 0.00	1.65 ± 0.03	0.19 ± 0.00	2.66 ± 0.14	0.28 ± 0.00	2.09 ± 0.01	0.23 ± 0.00
81	(*E*)-Caryophyllene	Sesquiterpene	1.0	995	1423	1424	32.03 ± 1.68	3.11 ± 0.02	251.13 ± 4.33	28.78 ± 0.03	130.88 ± 6.65	13.69 ± 0.19	220.88 ± 1.16	23.99 ± 0.02
82	γ-Elemene	Sesquiterpene	1.0	965	1432	1432	7.50 ± 0.40	0.73 ± 0.00	0.73 ± 0.03	0.08 ± 0.00	3.11 ± 0.12	0.33 ± 0.01	0.63 ± 0.01	0.07 ± 0.00
83	α-, (*E*)-Bergamotene	Sesquiterpene	1.0	994	1435	1432	1.85 ± 0.10	0.18 ± 0.00	9.02 ± 0.16	1.03 ± 0.00	21.38 ± 1.04	2.24 ± 0.04	15.83 ± 0.08	1.72 ± 0.00
84	α-Guaiene	Sesquiterpene	1.0	950	1438	1438	0.12 ± 0.01	0.01 ± 0.00	1.77 ± 0.03	0.20 ± 0.00	-	-	1.07 ± 0.02	0.12 ± 0.00
85	Aromadendrene	Sesquiterpene	1.0	986	1442	1438	-	-	0.62 ± 0.05	0.07 ± 0.01	-	-	-	-
86	Guaia-6,9-diene	Sesquiterpene	1.0	969	1444	1444	-	-	2.06 ± 0.09	0.24 ± 0.01	1.66 ± 0.09	0.17 ± 0.00	1.49 ± 0.01	0.16 ± 0.00
87	(*E*)-Geranylacetone	ketone	1.3	948	1450	1450	-	-	2.06 ± 0.04	0.18 ± 0.00	-	-	1.34 ± 0.02	0.09 ± 0.00
88	(*E*)-, β-Farnesene	Sesquiterpene	1.0	992	1454	1452	3.78 ± 0.19	0.37 ± 0.00	8.61 ± 0.15	0.99 ± 0.00	34.63 ± 1.54	3.62 ± 0.07	16.36 ± 0.09	1.78 ± 0.00
89	α-Humulene	Sesquiterpene	1.0	996	1458	1454	9.21 ± 0.48	0.89 ± 0.01	85.27 ± 1.55	9.77 ± 0.02	44.99 ± 2.08	4.71 ± 0.09	66.14 ± 0.35	7.18 ± 0.01
90	9-*epi*-(E)-Caryophyllene	Sesquiterpene	1.0	981	1463	1464	0.30 ± 0.02	0.03 ± 0.00	9.64 ± 0.17	1.10 ± 0.00	6.14 ± 0.31	0.64 ± 0.01	4.72 ± 0.03	0.51 ± 0.00
91	β-Acoradiene	Sesquiterpene	1.0	915	1471	1467	-	-			0.35 ± 0.02	0.04 ± 0.00	-	-
92	Drima-7,9(11)-diene	Sesquiterpene	1.0	980	1473	1473	-	-	1.23 ± 0.06	0.14 ± 0.01	0.71 ± 0.04	0.07 ± 0.01	-	-
93	Selina-4,11-diene	Sesquiterpene	1.0	983	1476	1476	-	-	1.05 ± 0.08	0.12 ± 0.01	1.13 ± 0.10	0.12 ± 0.00	1.31 ± 0.09	0.14 ± 0.01
94	β-Chamigrene	Sesquiterpene	1.0	959	1477	1479	-	-			-	-	-	-
95	γ-Muurolene	Sesquiterpene	1.0	981	1478	1478	0.54 ± 0.03	0.05 ± 0.00	0.93 ± 0.14	0.11 ± 0.01	-	-	-	-
96	α-Neocallitropsene	Sesquiterpene	1.0	938	1479	1480	0.28 ± 0.02	0.03 ± 0.00	-	-	1.73 ± 0.08	0.18 ± 0.00	1.12 ± 0.08	0.12 ± 0.01
97	γ-Gurjunene	Sesquiterpene	1.0	957	1480	1476	-	-	1.06 ± 0.05	0.12 ± 0.01	-	-	-	-
98	γ-Curcumene	Sesquiterpene	1.0	960	1480	1482	-	-	-	-	-	-	-	-
99	α-Amorphene	Sesquiterpene	1.0	963	1482	1482	-	-	5.25 ± 0.30	0.60 ± 0.03	1.19 ± 0.06	0.12 ± 0.00	-	-
100	α-Curcumene	Sesquiterpene	1.0	968	1483	1480	-	-	-	-	-	-	4.88 ± 0.03	0.53 ± 0.00
101	Aristolochene	Sesquiterpene	1.0	921	1487	1490	-	-	10.73 ± 0.16	1.23 ± 0.01	-	-	-	-
102	Eremophilene	Sesquiterpene	1.0	939	1486	1491	0.35 ± 0.02	0.03 ± 0.00	3.39 ± 0.07	0.39 ± 0.00	4.01 ± 0.19	0.42 ± 0.01	7.63 ± 0.15	0.83 ± 0.02
103	β-, (*E*)-Bergamotene	Sesquiterpene	1.0	935	1487	1483	-	-	-	-	1.44 ± 0.06	0.15 ± 0.00		
104	β-Selinene	Sesquiterpene	1.0	988	1492	1492	0.79 ± 0.04	0.08 ± 0.00	25.53 ± 0.47	2.93 ± 0.00	8.75 ± 0.34	0.92 ± 0.02	20.19 ± 0.16	2.19 ± 0.01
105	Valencene	Sesquiterpene	1.0	983	1489	1492	0.35 ± 0.02	0.03 ± 0.00	6.68 ± 0.12	0.77 ± 0.00	2.56 ± 0.15	0.27 ± 0.00	3.46 ± 0.06	0.38 ± 0.01
106	α-Selinene	Sesquiterpene	1.0	985	1499	1501	1.15 ± 0.06	0.11 ± 0.00	17.87 ± 0.32	2.05 ± 0.00	7.24 ± 0.32	0.76 ± 0.02	15.40 ± 0.07	1.67 ± 0.00
107	(*Z*)-, α-Bisabolene	Sesquiterpene	1.0	933	1502	1503	0.16 ± 0.01	0.02 ± 0.00	-	-	-	-	-	-
108	ε-Amorphene	Sesquiterpene	1.0	961	1503	1502	-	-	-	-	1.63 ± 0.07	0.17 ± 0.00	-	-
109	α-Bulnesene	Sesquiterpene	1.0	990	1505	1505	-	-	4.33 ± 0.08	0.50 ± 0.00	-	-	1.99 ± 0.06	0.22 ± 0.01
110	(*E*,*E*)-, α-Farnesene	Sesquiterpene	1.0	995	1506	1504	-	-	-	-	1.89 ± 0.08	0.20 ± 0.00	-	-
111	β-Bisabolene	Sesquiterpene	1.0	995	1510	1508	6.83 ± 0.34	0.66 ± 0.00	3.58 ± 0.09	0.41 ± 0.00	2.57 ± 0.11	0.27 ± 0.01	6.81 ± 0.03	0.74 ± 0.00
112	(*Z*)-, γ-Bisabolene	Sesquiterpene	1.0	952	1512	1511	-	-	0.84 ± 0.03	0.10 ± 0.00	1.48 ± 0.06	0.15 ± 0.00	-	-
113	Sesquicineole	Epoxide	1.5	912	1516	1514	0.73 ± 0.05	0.05 ± 0.00	-	-	-	-	1.54 ± 0.01	0.11 ± 0.00
114	γ-Cadinene	Sesquiterpene	1.0	974	1517	1512	-	-	2.28 ± 0.05	0.26 ± 0.00	0.64 ± 0.05	0.07 ± 0.00	-	-
115	δ-Cadinene	Sesquiterpene	1.0	967	1522	1518	-	-	-	-	-	-	-	-
116	β-Sesquiphellandrene	Sesquiterpene	1.0	991	1526	1523	0.31 ± 0.02	0.03 ± 0.00	0.68 ± 0.02	0.08 ± 0.00	0.75 ± 0.03	0.08 ± 0.00	0.47 ± 0.01	0.05 ± 0.00
117	Selina-4(15),7(11)-diene	Sesquiterpene	1.0	985	1541	1540	7.80 ± 1.12	0.75 ± 0.08	23.18 ± 0.40	2.66 ± 0.01	11.74 ± 0.46	1.23 ± 0.03	25.97 ± 0.12	2.82 ± 0.00
118	(*E*)-, α-Bisabolene	Sesquiterpene	1.0	953	1543	1540	8.45 ± 0.45	0.82 ± 0.07	-	-	-	-	-	-
119	Selina-3,7(11)-diene	Sesquiterpene	1.0	994	1546	1546	10.86 ± 0.56	1.05 ± 0.01	18.36 ± 0.32	2.10 ± 0.00	14.07 ± 0.53	1.47 ± 0.04	20.99 ± 0.10	2.28 ± 0.01
120	Germacrene B	Sesquiterpene	1.0	926	1552	1557	-	-	-	-	-	-	-	-
121	*epi*-Longipinanol	Alcohol	1.3	929	1554	1558	-	-	0.81 ± 0.02	0.07 ± 0.00	1.10 ± 0.06	0.09 ± 0.00	2.01 ± 0.03	0.17 ± 0.00
122	Longicamphenylone	ketone	1.3	896	1559	1560	-	-	3.60 ± 0.06	0.32 ± 0.00	-	-	-	-
123	(*E*)-Nerolidol	Alcohol	1.3	916	1563	1561	1.95 ± 0.13	0.15 ± 0.00	33.68 ± 0.59	2.97 ± 0.01	4.69 ± 0.14	0.38 ± 0.01	10.24 ± 0.03	0.86 ± 0.00
124	Longipinanol	Alcohol	1.3	948	1577	1572	-	-	2.56 ± 0.06	0.23 ± 0.00	-	-	1.68 ± 0.01	0.18 ± 0.00
125	Caryolan-8-ol	Alcohol	1.3	957	1580	1575	-	-	1.56 ± 0.05	0.14 ± 0.00	-	-	-	-
126	Caryophyllene oxide	Epoxide	1.5	991	1586	1587	0.71 ± 0.05	0.05 ± 0.00	94.97 ± 1.75	7.26 ± 0.02	21.89 ± 0.65	1.53 ± 0.05	61.70 ± 0.27	4.47 ± 0.01
127	Fokienol	Alcohol	1.3	915	1594	1596	-	-	1.43 ± 0.03	0.13 ± 0.00	1.02 ± 0.03	0.08 ± 0.00	1.10 ± 0.01	0.09 ± 0.00
128	Guaiol	Alcohol	1.3	993	1601	1600	9.25 ± 0.46	0.69 ± 0.00	-	-	-	-	-	-
129	Javanol isomer II	Alcohol	1.3	922	1610	1612	-	-	2.16 ± 0.05	0.19 ± 0.00	-	-	-	-
130	5-*epi*-7-*epi*-α-Eudesmol	Alcohol	1.3	956	1611	1610	0.56 ± 0.03	0.04 ± 0.00			-	-	-	-
131	Humulene epoxide II	Epoxide	1.5	991	1615	1613	-	-	28.56 ± 0.47	2.18 ± 0.01	7.39 ± 0.20	0.52 ± 0.02	18.90 ± 0.08	1.37 ± 0.00
132	*epi*-γ-Eudesmol	Alcohol	1.3	989	1627	1624	8.38 ± 0.42	0.63 ± 0.00	15.47 ± 0.24	1.36 ± 0.00	-	-	-	-
133	γ-Eudesmol	Alcohol	1.3	987	1628	1632	-	-	-	-	-	-	-	-
134	Eremoligenol	Alcohol	1.3	961	1636	1632	-	-	-	-	3.84 ± 0.10	0.31 ± 0.01	5.75 ± 0.03	0.48 ± 0.00
135	Caryophylla-4(12),8(13)-dien-5-β-ol	Alcohol	1.3	909	1639	1636	-	-	15.22 ± 1.02	1.34 ± 0.07	-	-	5.15 ± 0.03	0.43 ± 0.00
136	Caryophylla-4(12),8(13)-dien-5-α-ol	Alcohol	1.3	968	1643	1642	-	-	11.38 ± 0.19	1.00 ± 0.00	2.45 ± 0.06	0.20 ± 0.01	-	-
137	Agarospirol	Alcohol	1.3	958	1645	1646	0.31 ± 0.02	0.02 ± 0.00	-	-	-	-	-	-
138	Hinesol	Alcohol	1.3	974	1645	1645	-	-	-	-	-	-	-	-
139	*allo*-Aromandendrene epoxide	Epoxide	1.5	946	1649	1644	-	-	1.48 ± 0.03	0.11 ± 0.00	-	-	-	-
140	Pogostol	Alcohol	1.3	896	1649	1650	0.81 ± 0.04	0.06 ± 0.00	-	-	-	-	-	-
141	β-Eudesmol	Alcohol	1.3	933	1661	1656	8.35 ± 0.41	0.62 ± 0.00	-	-	-	-	-	-
142	14-hydroxy-(*Z*)-Caryophyllene	Alcohol	1.3	951	1662	1664	-	-	17.81 ± 0.30	1.57 ± 0.01	1.71 ± 0.05	0.14 ± 0.01	8.13 ± 0.03	0.68 ± 0.00
143	*neo*-Intermedeol	Alcohol	1.3	932	1662	1661	-	-	-	-	-	-	-	-
144	7-*epi*-α-Eudesmol	Alcohol	1.3	960	1667	1665	0.31 ± 0.01	0.02 ± 0.00	-	-	-	-	-	-
145	Intermedeol	Alcohol	1.3	967	1670	1668	-	-	1.86 ± 0.05	0.16 ± 0.00	-	-	1.49 ± 0.01	0.12 ± 0.00
146	Bulnesol	Alcohol	1.3	991	1670	1670	5.83 ± 0.28	0.44 ± 0.00	-	-	-	-	-	-
147	Khusinol	Alcohol	1.3	918	1677	1677	-	-	14.11 ± 0.23	1.24 ± 0.00	2.02 ± 0.03	0.16 ± 0.01	7.02 ± 0.05	0.59 ± 0.00
148	α-Bisabolol	Alcohol	1.3	983	1689	1688	14.01 ± 0.65	1.05 ± 0.01	6.83 ± 0.10	0.60 ± 0.00	1.16 ± 0.03	0.09 ± 0.00	13.80 ± 0.02	1.15 ± 0.00
149	Juniper camphor	Alcohol	1.3	928	1701	1696	0.30 ± 0.03	0.02 ± 0.00	-	-	-	-	-	-
150	Caryophyllene acetate isomer I	Ester	1.6	917	1704	1701	-	-	6.72 ± 0.11	0.48 ± 0.00	1.71 ± 0.02	0.11 ± 0.01	2.64 ± 0.02	0.18 ± 0.00
151	*iso*-Longifolol	Alcohol	1.3	933	1726	1727	-	-	-	-	-	-	0.95 ± 0.01	0.08 ± 0.00
152	Nootkatone	Ketone	1.3	974	1810	1806	-	-	-	-	-	-	0.77 ± 0.01	0.06 ± 0.00
153	Phytone	Ketone	1.3	991	1843	1841	-	-	2.69 ± 0.04	0.24 ± 0.00	-	-	2.42 ± 0.01	0.20 ± 0.00
154	*m*-Camphorene	Diterpene	1.0	979	1950	1946	0.31 ± 0.01	0.03 ± 0.00	0.67 ± 0.01	0.08 ± 0.00	0.22 ± 0.01	0.02 ± 0.00	0.52 ± 0.01	0.06 ± 0.00
155	*p*-Camphorene	Diterpene	1.0	983	1986	1984	-	-	0.54 ± 0.01	0.06 ± 0.00	-	-	0.35 ± 0.00	0.04 ± 0.00
156	Phytol	Alcohol	1.3	956	2111	2111	-	-	-	-	-	-	2.33 ± 0.03	0.20 ± 0.00
157	Cannabidivarin (CBDV)	Cannabinoid	1.0	897	2215	2216	-	-	0.55 ± 0.01	0.05 ± 0.00	-	-	-	-
158	Cannabicitran (CBT)	Cannabinoid	1.0	954	2281	2284	-	-	1.19 ± 0.02	0.11 ± 0.00	-	-	2.60 ± 0.36	0.13 ± 0.00
159	Cannabicyclol (CBL)	Cannabinoid	1.0	890	2373	2374	-	-	0.18 ± 0.01	0.02 ± 0.00	-	-	-	-
160	Cannabidiol (CBD)	Cannabinoid	1.0	983	2424	2421	2.11 ± 0.06	0.15 ± 0.00	19.77 ± 0.22	1.75 ± 0.03	5.33 ± 0.28	0.16 ± 0.01	8.90 ± 1.20	0.44 ± 0.00
161	Cannabichromene (CBC)	Cannabinoid	1.0	892	2433	2435	-	-	0.50 ± 0.01	0.04 ± 0.00	-	-	0.41 ± 0.05	0.02 ± 0.00
162	δ8-Tetrahydrocannabinol (Δ^8^-THC)	Cannabinoid	1.0	890	2501	2501	-	-	0.12 ± 0.01	0.01 ± 0.00	-	-	0.28 ± 0.04	0.01 ± 0.00
163	δ9-Tetrahydrocannabinol (Δ^9^-THC)	Cannabinoid	1.0	910	2525	2527	-	-	0.18 ± 0.00	0.02 ± 0.00	-	-	0.56 ± 0.08	0.03 ± 0.00
164	*n*-Heptacosane	Aliphatic	1.0	953	2701	2700	-	-	0.30 ± 0.01	0.03 ± 0.00	-	-	0.26 ± 0.01	0.03 ± 0.00
165	*n*-Nonacosane	Aliphatic	1.0	958	2902	2900	-	-	0.30 ± 0.01	0.03 ± 0.00	-	-	0.29 ± 0.00	0.03 ± 0.00
	*NOT IDENTIFIED*	-	-	-	-	-	12.26 ± 0.37	1.21 ± 0.00	78.47 ± 1.13	8.99 ± 0.08	2.78 ± 0.01	0.30 ± 0.00	63.26 ± 0.80	6.87 ± 0.08
	*TOTAL*	-	-	-	-	-	946.09 ± 60.65	100.00	963.70 ± 14.06	100.00 ± 0.00	-	-	973.20 ± 5.05	100.00 ± 0.00
	*HYDROCARBON Compounds*	-	-	-	-	-	851.75 ± 59.81	91.41 ± 0.03	563.45 ± 8.66	64.35 ± 0.05	0.53 ± 0.01	0.06 ± 0.00	733.05 ± 5.06	79.77 ± 0.09
	*Monoterpenes*	-	-	-	-	-	758.22 ± 64.53	82.33 ± 0.07	53.86 ± 0.55	5.95 ± 0.04			285.80 ± 6.01	31.19 ± 0.15
	*Sesquiterpenes*	-	-	-	-	-	93.23 ± 4.83	9.05 ± 0.05	507.78 ± 9.11	58.19 ± 0.01	181.89 ± 0.64	19.50 ± 0.32	445.83 ± 2.18	48.43 ± 0.07
	*Diterpenes*	-	-	-	-	-	0.31 ± 0.01	0.03 ± 0.00	1.21 ± 0.02	0.14 ± 0.00	0.20 ± 0.01	0.02 ± 0.00	0.87 ± 0.01	0.09 ± 0.00
	*Aliphatic*	-	-	-	-	-	0.00 ± 0.00	0.00 ± 0.00	0.60 ± 0.01	0.07 ± 0.00	3.35 ± 0.01	0.36 ± 0.01	0.55 ± 0.01	0.06 ± 0.00
	*OXIGENATED Compounds*	-	-	-	-	-	94.34 ± 0.92	7.38 ± 0.02	321.78 ± 4.39	26.66 ± 0.06	0.72 ± 0.00	0.08 ± 0.00	176.90 ± 1.08	13.36 ± 0.01
	*Aldehydes*	-	-	-	-	-	0.29 ± 0.04	0.02 ± 0.00	1.46 ± 0.03	0.12 ± 0.00	54.17 ± 0.12	5.81 ± 0.09	0.62 ± 0.02	0.05 ± 0.00
	*Alcohols*	-	-	-	-	-	88.32 ± 0.85	6.93 ± 0.02	154.84 ± 2.23	13.55 ± 0.07			71.01 ± 0.41	5.94 ± 0.01
	*Esters*	-	-	-	-	-	0.69 ± 0.06	0.05 ± 0.00	7.16 ± 0.11	0.51 ± 0.00	143.12 ± 0.37	15.34 ± 0.23	4.72 ± 0.11	0.32 ± 0.01
	*Ketones*	-	-	-	-	-	1.51 ± 0.13	0.13 ± 0.00	10.19 ± 0.06	0.89 ± 0.01			4.97 ± 0.05	0.42 ± 0.00
	*Epoxides*	-	-	-	-	-	1.44 ± 0.09	0.09 ± 0.00	125.65 ± 2.22	9.60 ± 0.02			82.84 ± 0.34	6.00 ± 0.01
	*Cannabinoids*	-	-	-	-	-	2.11 ± 0.06	0.15 ± 0.00	22.48 ± 0.23	1.99 ± 0.03	2.13 ± 0.02	0.23 ± 0.01	12.74 ± 1.73	0.63 ± 0.01
	*Distillation Yield*	-	-	-	-	-	0.274		0.015		0.148		0.035	

**Table 2:** List of volatile compounds detected in cannabis essential oils (EOs) distilled from inflorescences of Carmagnola (fresh), Felina 32 (dried) and Finola (fresh) cultivars. Abbreviations: R.F.: response factor; match: database spectral matching; LRI_exp_: experimental LRI; LRI_ref_: reference LRI. Quantitative results are expressed both in absolute manner (mg g^−1^) and in relative percentages (%). The compounds are also grouped in monoterpene, sesquiterpene, diterpene and aliphatic hydrocarbons and as well as oxygenated compounds including aldehyde, alcohol, ester, ketone, epoxide, and cannabinoid. The cannabis EO yields are also reported.

ID	Name	Chemical Subclass	R.F.	Match	LRI_exp_	LRI_ref_	Fresh Carmagnola		Dried Felina 32		Fresh Finola	
							mg g^−1^	%	mg g^−1^	%	mg g^−1^	%
1	Hashishene	Monoterpene	1.0	947	921	921	0.44 ± 0.01	0.05 ± 0.00	2.21 ± 0.05	0.24 ± 0.00	1.70 ± 0.07	0.17 ± 0.00
2	Tricyclene	Monoterpene	1.0	931	924	923	*-*	*-*	*-*	*-*	*-*	*-*
3	α-Thujene	Monoterpene	1.0	991	927	927	0.31 ± 0.01	0.03 ± 0.00	0.24 ± 0.01	0.03 ± 0.00	*-*	*-*
4	Thuja-2,4(10)-diene	Monoterpene	1.0	950	954	953	*-*	*-*	73.31 ± 1.60	8.08 ± 0.03	*-*	*-*
5	α-Pinene	Monoterpene	1.0	980	935	933	5.91 ± 0.07	0.65 ± 0.01	0.22 ± 0.01	0.02 ± 0.00	28.40 ± 1.23	2.93 ± 0.02
6	α-Fenchene	Monoterpene	1.0	976	951	950	*-*	*-*	2.46 ± 0.06	0.27 ± 0.00	*-*	*-*
7	Camphene	Monoterpene	1.0	995	951	953	1.51 ± 0.02	0.17 ± 0.00	0.16 ± 0.01	0.02 ± 0.00	1.10 ± 0.04	0.11 ± 0.00
8	Sabinene	Monoterpene	1.0	886	974	972	*-*	*-*			*-*	*-*
9	β-Pinene	Monoterpene	1.0	986	980	978	10.19 ± 0.10	1.12 ± 0.01	17.02 ± 0.36	1.88 ± 0.01	11.52 ± 0.51	1.19 ± 0.01
10	6-methyl-Hept-5-en-2-one	Ketone	1.3	920	986	986	*-*	*-*	0.38 ± 0.01	0.03 ± 0.00	0.70 ± 0.03	0.06 ± 0.00
11	Myrcene	Monoterpene	1.0	982	993	991	299.43 ± 2.28	32.81 ± 0.19	16.90 ± 0.36	1.86 ± 0.01	54.73 ± 2.43	5.64 ± 0.03
12	*m*-Mentha-1(7), 8-diene	Monoterpene	1.0	873	1002	1001	0.20 ± 0.01	0.02 ± 0.00	*-*	*-*	*-*	*-*
13	*n*-Octanal	Aldehyde	1.3	975	1006	1006	*-*	*-*	*-*	*-*	0.65 ± 0.03	0.05 ± 0.00
14	α-Phellandrene	Monoterpene	1.0	978	1008	1007	0.12 ± 0.02	0.01 ± 0.00	0.64 ± 0.01	0.07 ± 0.00	*-*	*-*
15	δ3-Carene	Monoterpene	1.0	986	1011	1009	*-*	*-*	1.04 ± 0.02	0.11 ± 0.00	*-*	*-*
16	hexyl-Acetate	Ester	1.6	981	1013	1012	0.37 ± 0.02	0.03 ± 0.00	*-*	*-*	*-*	*-*
17	α-Terpinene	Monoterpene	1.0	983	1019	1018	1.02 ± 0.01	0.11 ± 0.00	0.84 ± 0.01	0.09 ± 0.00	0.11 ± 0.01	0.01 ± 0.00
18	*p*-Cymene	Monoterpene	1.0	963	1026	1025	0.91 ± 0.01	0.10 ± 0.00	3.28 ± 0.06	0.36 ± 0.00	0.41 ± 0.02	0.03 ± 0.00
19	Limonene	Monoterpene	1.0	991	1031	1030	99.54 ± 0.83	10.90 ± 0.06	11.62 ± 0.25	1.28 ± 0.00	20.18 ± 0.91	2.08 ± 0.01
20	β-Phellandrene	Monoterpene	1.0	975	1032	1031	*-*	*-*	*-*	*-*	*-*	*-*
21	Eucalyptol	Alcohol	1.3	984	1034	1032	14.47 ± 0.13	1.22 ± 0.01	1.69 ± 0.03	0.14 ± 0.00	*-*	*-*
22	(*Z*)-, β-Ocimene	Monoterpene	1.0	951	1036	1035	0.19 ± 0.01	0.02 ± 0.00	3.91 ± 0.08	0.43 ± 0.00	0.45 ± 0.02	0.05 ± 0.00
23	(*E*)-, β-Ocimene	Monoterpene	1.0	980	1047	1046	*-*	*-*	2.46 ± 0.05	0.27 ± 0.00	0.13 ± 0.01	0.01 ± 0.00
24	γ-Terpinene	Monoterpene	1.0	985	1060	1058	2.78 ± 0.02	0.30 ± 0.00	1.06 ± 0.02	0.12 ± 0.00	0.20 ± 0.01	0.02 ± 0.00
25	(*Z*)-Sabinene hydrate	Alcohol	1.3	963	1072	1069	0.74 ± 0.16	0.06 ± 0.01	0.65 ± 0.01	0.06 ± 0.00	*-*	*-*
26	Octanol	Alcohol	1.3	920	1072	1076	1.11 ± 0.16	0.09 ± 0.01	*-*	*-*	3.45 ± 0.15	0.27 ± 0.00
27	Terpinolene	Monoterpene	1.0	992	1088	1086	1.70 ± 0.01	0.19 ± 0.00	3.29 ± 0.06	0.36 ± 0.00	0.38 ± 0.02	0.04 ± 0.00
28	Fenchone	Ketone	1.3	990	1091	1090	1.35 ± 0.02	0.11 ± 0.00	*-*	*-*	2.40 ± 0.11	0.19 ± 0.00
29	*p*-Cymenene	Monoterpene	1.0	964	1093	1093	*-*	*-*	1.66 ± 0.03	0.18 ± 0.00	*-*	*-*
30	Linalool	Alcohol	1.3	994	1102	1101	63.25 ± 0.38	5.33 ± 0.02	2.00 ± 0.04	0.17 ± 0.00	7.72 ± 0.37	0.61 ± 0.00
31	(*E*)-Sabinene hydrate	Alcohol	1.3	976	1104	1099	0.52 ± 0.07	0.04 ± 0.01	0.43 ± 0.01	0.04 ± 0.00	*-*	*-*
32	*n*-Nonanal	Aldehyde	1.3	972	1107	1107	1.01 ± 0.08	0.08 ± 0.01	1.19 ± 0.01	0.10 ± 0.00	2.58 ± 0.13	0.20 ± 0.00
33	Fenchyl alcohol	Alcohol	1.3	996	1122	1123	6.92 ± 0.04	0.58 ± 0.00	2.85 ± 0.05	0.24 ± 0.00	16.05 ± 0.74	1.27 ± 0.00
34	(*E*)-, *p*-Mentha-2,8-dien-1-ol	Alcohol	1.3	954	1125	1122	*-*	*-*	*-*	*-*	0.13 ± 0.00	0.01 ± 0.00
35	(*Z*)-, *p*-Menth-2-en-1-ol	Alcohol	1.3	915	1129	1124	4.08 ± 0.03	0.34 ± 0.00	1.95 ± 0.05	0.17 ± 0.00	11.58 ± 0.52	0.92 ± 0.00
36	*allo*-Ocim-(4*E*,6*Z*)-ene	Monoterpene	1.0	977	1130	1128	*-*	*-*	*-*	*-*	*-*	*-*
37	Limona ketone	Ketone	1.3	967	1128	1131	*-*	*-*	*-*	*-*	1.06 ± 0.02	0.08 ± 0.00
38	(*Z*)-, *p*-Mentha-2,8-dien-1-ol	Alcohol	1.3	980	1139	1138	*-*	*-*	0.82 ± 0.01	0.07 ± 0.00	0.99 ± 0.04	0.08 ± 0.00
39	(*E*)-Myroxide	Epoxide	1.5	973	1141	1141	*-*	*-*	*-*	*-*	*-*	*-*
40	(*E*)-Pinocarveol	Alcohol	1.3	992	1144	1141	*-*	*-*	2.31 ± 0.03	0.20 ± 0.00	*-*	*-*
41	Ipsdienol	Alcohol	1.3	985	1145	1146	1.76 ± 0.02	0.15 ± 0.00	*-*	*-*	0.58 ± 0.03	0.05 ± 0.00
42	Myrcenone	Ketone	1.3	924	1148	1149	0.67 ± 0.02	0.06 ± 0.00	*-*	*-*	*-*	*-*
43	*neo*-Isopulegol	Alcohol	1.3	914	1150	1148	0.37 ± 0.01	0.03 ± 0.00	*-*	*-*	1.37 ± 0.07	0.11 ± 0.00
44	(*Z*)-Pinene hydrate	Alcohol	1.3	943	1149	1144	*-*	*-*	*-*	*-*	*-*	*-*
45	Camphene hydrate	Alcohol	1.3	977	1158	1156	0.30 ± 0.01	0.03 ± 0.00	*-*	*-*	1.64 ± 0.08	0.13 ± 0.00
46	(*E*)-Verbenol	Alcohol	1.3	940	1149	1145	*-*	*-*	0.35 ± 0.00	0.03 ± 0.00	*-*	*-*
47	β-Pinene oxide	Epoxide	1.5	926	1153	1156	*-*	*-*	*-*	*-*	*-*	*-*
48	Camphene hydrate	Alcohol	1.3	910	1158	1156	*-*	*-*	0.27 ± 0.00	0.02 ± 0.00	*-*	*-*
49	Menthone	ketone	1.3	981	1159	1158	*-*	*-*	*-*	*-*	*-*	*-*
50	(*E*)-Pinocamphone	ketone	1.3	934	1163	1160	*-*	*-*	0.28 ± 0.00	0.02 ± 0.00	*-*	*-*
51	Pinocarvone	ketone	1.3	981	1166	1164	*-*	*-*	0.77 ± 0.01	0.06 ± 0.00	*-*	*-*
52	Borneol	Alcohol	1.3	998	1175	1173	2.74 ± 0.03	0.23 ± 0.00	1.79 ± 0.03	0.15 ± 0.00	4.69 ± 0.22	0.37 ± 0.00
53	Menthol	Alcohol	1.3	986	1180	1184	*-*	*-*	*-*	*-*	*-*	*-*
54	Terpinen-4-ol	Alcohol	1.3	982	1183	1184	1.98 ± 0.02	0.17 ± 0.00	0.93 ± 0.01	0.08 ± 0.00	0.49 ± 0.02	0.04 ± 0.00
55	*p*-Cymen-8-ol	Alcohol	1.3	913	1190	1189	*-*	*-*	1.08 ± 0.02	0.09 ± 0.00	*-*	*-*
56	hexyl-Butyrate	Ester	1.6	988	1193	1195	0.78 ± 0.02	0.05 ± 0.00	*-*	*-*	*-*	*-*
57	α-Terpineol	Alcohol	1.3	993	1198	1195	8.85 ± 0.07	0.75 ± 0.00	1.59 ± 0.03	0.14 ± 0.00	10.68 ± 0.51	0.85 ± 0.00
58	Myrtenol	Alcohol	1.3	860	1200	1202	*-*	*-*	1.08 ± 0.01	0.09 ± 0.00	*-*	*-*
59	octyl-Acetate	Ester	1.6	970	1212	1214	0.63 ± 0.03	0.04 ± 0.00	*-*	*-*	*-*	*-*
60	(*E*)-Piperitol	Alcohol	1.3	936	1213	1209	*-*	*-*	*-*	*-*	*-*	*-*
61	*endo*-Fenchyl acetate	Ester	1.6	897	1220	1221	*-*	*-*	*-*	*-*	0.20 ± 0.00	0.01 ± 0.00
62	(*E*)-Carveol	Alcohol	1.3	934	1223	1223	*-*	*-*	0.28 ± 0.00	0.02 ± 0.00	0.25 ± 0.02	0.02 ± 0.00
63	Citronellol	Alcohol	1.3	992	1229	1232	*-*	*-*	0.34 ± 0.00	0.03 ± 0.00	3.10 ± 0.16	0.25 ± 0.00
64	Dec-(4*Z*)-en-1-ol	Alcohol	1.3	976	1256	1258	1.03 ± 0.01	0.09 ± 0.00	*-*	*-*	0.83 ± 0.04	0.07 ± 0.00
65	Dec-(4*E*)-en-1-ol	Alcohol	1.3	994	1259	1262	*-*	*-*	*-*	*-*	1.88 ± 0.10	0.15 ± 0.00
66	Decyl alcohol	Alcohol	1.3	952	1275	1278	*-*	*-*	*-*	*-*	0.41 ± 0.02	0.03 ± 0.00
67	Bornyl Acetate	Ester	1.6	975	1286	1285	0.18 ± 0.00	0.01 ± 0.00	0.36 ± 0.01	0.03 ± 0.00	0.54 ± 0.02	0.03 ± 0.00
68	Cogeijerene	Sesquiterpene	1.0	920	1288	1286	*-*	*-*	0.17 ± 0.01	0.02 ± 0.00	*-*	*-*
69	*n*-Tridecane	Aliphatic	1.0	950	1301	1300	*-*	*-*	0.30 ± 0.01	0.03 ± 0.00	*-*	*-*
70	α-Cubebene	Sesquiterpene	1.0	968	1350	1347	*-*	*-*	*-*	*-*	0.22 ± 0.01	0.02 ± 0.00
71	Eugenol	Alcohol	1.3	933	1356	1357	*-*	*-*	0.72 ± 0.01	0.06 ± 0.00	*-*	*-*
72	α-Ylangene	Sesquiterpene	1.0	964	1372	1371	*-*	*-*	2.83 ± 0.08	0.32 ± 0.00	0.50 ± 0.01	0.05 ± 0.00
73	α-Copaene	Sesquiterpene	1.0	956	1372	1375	*-*	*-*	0.71 ± 0.04	0.08 ± 0.00	0.49 ± 0.01	0.05 ± 0.00
74	hexyl-Hexanoate	Ester	1.6	985	1387	1390	1.07 ± 0.01	0.07 ± 0.00	*-*	*-*	0.32 ± 0.01	0.02 ± 0.00
75	7-*epi*-Sesquithujene	Sesquiterpene	1.0	942	1389	1387	*-*	*-*	0.12 ± 0.01	0.01 ± 0.00	0.54 ± 0.01	0.06 ± 0.00
76	Sativene	Sesquiterpene	1.0	905	1397	1394	*-*	*-*	0.86 ± 0.04	0.10 ± 0.00	1.54 ± 0.02	0.16 ± 0.00
77	α-Funebrene	Sesquiterpene	1.0	909	1404	1403	0.26 ± 0.02	0.03 ± 0.00	0.34 ± 0.01	0.04 ± 0.00	0.29 ± 0.01	0.03 ± 0.00
78	(*Z*)-Caryophyllene	Sesquiterpene	1.0	953	1407	1413	0.28 ± 0.01	0.03 ± 0.00	5.37 ± 0.13	0.60 ± 0.00	1.39 ± 0.01	0.15 ± 0.00
79	α-Gurjunene	Sesquiterpene	1.0	986	1410	1406	0.28 ± 0.01	0.03 ± 0.00	0.73 ± 0.02	0.08 ± 0.00	*-*	*-*
80	α-, (*Z*)-Bergamotene	Sesquiterpene	1.0	978	1415	1416	2.07 ± 0.10	0.23 ± 0.00	2.38 ± 0.06	0.27 ± 0.00	0.75 ± 0.01	0.08 ± 0.00
81	(*E*)-Caryophyllene	Sesquiterpene	1.0	995	1423	1424	132.31 ± 6.31	14.57 ± 0.02	236.88 ± 5.47	26.50 ± 0.01	290.18 ± 3.69	30.66 ± 0.02
82	γ-Elemene	Sesquiterpene	1.0	965	1432	1432	*-*	*-*	0.63 ± 0.01	0.07 ± 0.00	0.71 ± 0.02	0.08 ± 0.00
83	α-, (*E*)-Bergamotene	Sesquiterpene	1.0	994	1435	1432	9.03 ± 0.42	0.99 ± 0.00	14.02 ± 0.33	1.57 ± 0.00	11.83 ± 0.12	1.25 ± 0.00
84	α-Guaiene	Sesquiterpene	1.0	950	1438	1438	0.49 ± 0.02	0.05 ± 0.00	0.72 ± 0.02	0.08 ± 0.00	38.25 ± 0.46	4.04 ± 0.01
85	Aromadendrene	Sesquiterpene	1.0	986	1442	1438	0.34 ± 0.02	0.04 ± 0.00	*-*	*-*	*-*	*-*
86	Guaia-6,9-diene	Sesquiterpene	1.0	969	1444	1444	*-*	*-*	2.42 ± 0.08	0.27 ± 0.00	0.20 ± 0.01	0.02 ± 0.00
87	(*E*)-Geranylacetone	ketone	1.3	948	1450	1450	*-*	*-*	2.08 ± 0.01	0.18 ± 0.00	*-*	*-*
88	(*E*)-, β-Farnesene	Sesquiterpene	1.0	992	1454	1452	16.48 ± 0.74	1.82 ± 0.01	9.57 ± 0.23	1.07 ± 0.00	9.44 ± 0.11	1.00 ± 0.00
89	α-Humulene	Sesquiterpene	1.0	996	1458	1454	39.07 ± 1.81	4.30 ± 0.01	78.88 ± 1.82	8.82 ± 0.00	77.90 ± 0.96	8.23 ± 0.01
90	9-*epi*-(*E*)-Caryophyllene	Sesquiterpene	1.0	981	1463	1464	2.84 ± 0.13	0.31 ± 0.00	10.22 ± 0.24	1.14 ± 0.00	1.13 ± 0.01	0.12 ± 0.00
91	β-Acoradiene	Sesquiterpene	1.0	915	1471	1467	*-*	*-*	0.31 ± 0.01	0.04 ± 0.00	0.61 ± 0.01	0.06 ± 0.00
92	Drima-7,9(11)-diene	Sesquiterpene	1.0	980	1473	1473	*-*	*-*	0.92 ± 0.04	0.10 ± 0.00	*-*	*-*
93	Selina-4,11-diene	Sesquiterpene	1.0	983	1476	1476	*-*	*-*	1.09 ± 0.06	0.12 ± 0.01	*-*	*-*
94	β-Chamigrene	Sesquiterpene	1.0	959	1477	1479	*-*	*-*	*-*	*-*	4.45 ± 0.05	0.47 ± 0.00
95	γ-Muurolene	Sesquiterpene	1.0	981	1478	1478	*-*	*-*	0.44 ± 0.01	0.05 ± 0.00	*-*	*-*
96	α-Neocallitropsene	Sesquiterpene	1.0	938	1479	1480	*-*	*-*	1.51 ± 0.13	0.17 ± 0.01	*-*	*-*
97	γ-Gurjunene	Sesquiterpene	1.0	957	1480	1476	*-*	*-*	*-*	*-*	2.19 ± 0.02	0.23 ± 0.00
98	γ-Curcumene	Sesquiterpene	1.0	960	1480	1482	0.52 ± 0.02	0.06 ± 0.00	4.96 ± 0.12	0.55 ± 0.00	*-*	*-*
99	α-Amorphene	Sesquiterpene	1.0	963	1482	1482	*-*	*-*	*-*	*-*	*-*	*-*
100	α-Curcumene	Sesquiterpene	1.0	968	1483	1480	0.32 ± 0.02	0.04 ± 0.00	*-*	*-*	0.94 ± 0.01	0.10 ± 0.00
101	Aristolochene	Sesquiterpene	1.0	921	1487	1490	*-*	*-*	*-*	*-*	*-*	*-*
102	Eremophilene	Sesquiterpene	1.0	939	1486	1491	*-*	*-*	8.44 ± 0.18	0.94 ± 0.00	6.39 ± 0.05	0.67 ± 0.00
103	β-, (*E*)-Bergamotene	Sesquiterpene	1.0	935	1487	1483	0.63 ± 0.03	0.07 ± 0.00	*-*	*-*	*-*	*-*
104	β-Selinene	Sesquiterpene	1.0	988	1492	1492	0.91 ± 0.04	0.10 ± 0.00	22.04 ± 0.62	2.46 ± 0.01	12.19 ± 0.16	1.29 ± 0.00
105	Valencene	Sesquiterpene	1.0	983	1489	1492	0.47 ± 0.02	0.05 ± 0.00	4.98 ± 0.08	0.56 ± 0.00	1.52 ± 0.01	0.16 ± 0.00
106	α-Selinene	Sesquiterpene	1.0	985	1499	1501	0.80 ± 0.03	0.09 ± 0.00	15.55 ± 0.30	1.74 ± 0.01	13.68 ± 0.16	1.45 ± 0.00
107	(*Z*)-, α-Bisabolene	Sesquiterpene	1.0	933	1502	1503	0.54 ± 0.03	0.06 ± 0.00	*-*	*-*	2.11 ± 0.23	0.22 ± 0.02
108	ε-Amorphene	Sesquiterpene	1.0	961	1503	1502	*-*	*-*	0.68 ± 0.05	0.08 ± 0.00	*-*	*-*
109	α-Bulnesene	Sesquiterpene	1.0	990	1505	1505	*-*	*-*	1.28 ± 0.01	0.14 ± 0.00	75.38 ± 1.10	7.97 ± 0.03
110	(*E*,*E*)-, α-Farnesene	Sesquiterpene	1.0	995	1506	1504	5.71 ± 0.23	0.63 ± 0.01	*-*	*-*	*-*	*-*
111	β-Bisabolene	Sesquiterpene	1.0	995	1510	1508	13.51 ± 0.57	1.49 ± 0.01	2.52 ± 0.06	0.28 ± 0.00	17.46 ± 0.25	1.85 ± 0.02
112	(*Z*)-, γ-Bisabolene	Sesquiterpene	1.0	952	1512	1511	*-*	*-*	1.04 ± 0.04	0.12 ± 0.00	*-*	*-*
113	Sesquicineole	Epoxide	1.5	912	1516	1514	3.28 ± 0.14	0.24 ± 0.00	*-*	*-*	3.24 ± 0.05	0.23 ± 0.00
114	γ-Cadinene	Sesquiterpene	1.0	974	1517	1512	*-*	*-*	*-*	*-*	*-*	*-*
115	δ-Cadinene	Sesquiterpene	1.0	967	1522	1518	0.37 ± 0.01	0.04 ± 0.00	1.19 ± 0.10	0.13 ± 0.01	*-*	*-*
116	β-Sesquiphellandrene	Sesquiterpene	1.0	991	1526	1523	2.23 ± 0.09	0.25 ± 0.00	0.56 ± 0.02	0.06 ± 0.00	1.32 ± 0.01	0.14 ± 0.00
117	Selina-4(15),7(11)-diene	Sesquiterpene	1.0	985	1541	1540	*-*	*-*	18.09 ± 0.40	2.02 ± 0.00	33.04 ± 1.28	3.49 ± 0.17
118	(*E*)-, α-Bisabolene	Sesquiterpene	1.0	953	1543	1540	13.00 ± 0.55	1.43 ± 0.01	*-*	*-*	30.14 ± 1.91	3.18 ± 0.17
119	Selina-3,7(11)-diene	Sesquiterpene	1.0	994	1546	1546	0.55 ± 0.03	0.06 ± 0.00	14.21 ± 0.34	1.59 ± 0.00	40.18 ± 0.48	4.25 ± 0.00
120	Germacrene B	Sesquiterpene	1.0	926	1552	1557	0.36 ± 0.01	0.04 ± 0.00	*-*	*-*	*-*	*-*
121	*epi*-Longipinanol	Alcohol	1.3	929	1554	1558	0.55 ± 0.03	0.05 ± 0.00	3.18 ± 0.06	0.27 ± 0.00	*-*	*-*
122	Longicamphenylone	ketone	1.3	896	1559	1560	*-*	*-*	*-*	*-*	*-*	*-*
123	(*E*)-Nerolidol	Alcohol	1.3	916	1563	1561	10.53 ± 0.40	0.89 ± 0.01	22.99 ± 0.52	1.98 ± 0.00	5.31 ± 0.05	0.43 ± 0.00
124	Longipinanol	Alcohol	1.3	948	1577	1572	0.34 ± 0.02	0.04 ± 0.00	2.23 ± 0.04	0.19 ± 0.00	0.49 ± 0.10	0.04 ± 0.00
125	Caryolan-8-ol	Alcohol	1.3	957	1580	1575	*-*	*-*	*-*	*-*	*-*	*-*
126	Caryophyllene oxide	Epoxide	1.5	991	1586	1587	4.26 ± 0.18	0.31 ± 0.00	88.75 ± 2.03	6.62 ± 0.00	21.64 ± 0.28	1.52 ± 0.00
127	Fokienol	Alcohol	1.3	915	1594	1596	*-*	*-*	*-*	*-*	*-*	*-*
128	Guaiol	Alcohol	1.3	993	1601	1600	34.95 ± 1.31	2.96 ± 0.04	*-*	*-*	*-*	*-*
129	Javanol isomer II	Alcohol	1.3	922	1610	1612	*-*	*-*	*-*	*-*	*-*	*-*
130	5-*epi*-7-*epi*-α-Eudesmol	Alcohol	1.3	956	1611	1610	2.71 ± 0.10	0.23 ± 0.00	*-*	*-*	*-*	*-*
131	Humulene epoxide II	Epoxide	1.5	991	1615	1613	1.44 ± 0.07	0.11 ± 0.00	27.35 ± 0.58	2.04 ± 0.00	6.26 ± 0.09	0.44 ± 0.00
132	*epi*-γ-Eudesmol	Alcohol	1.3	989	1627	1624	*-*	*-*	13.81 ± 0.33	1.19 ± 0.00	*-*	*-*
133	γ-Eudesmol	Alcohol	1.3	987	1628	1632	31.75 ± 1.20	2.69 ± 0.03	*-*	*-*	*-*	*-*
134	Eremoligenol	Alcohol	1.3	961	1636	1632	6.47 ± 0.25	0.55 ± 0.01	*-*	*-*	*-*	*-*
135	Caryophylla-4(12),8(13)-dien-5-β-ol	Alcohol	1.3	909	1639	1636	*-*	*-*	*-*	*-*	*-*	*-*
136	Caryophylla-4(12),8(13)-dien-5-α-ol	Alcohol	1.3	968	1643	1642	*-*	*-*	15.06 ± 0.36	1.30 ± 0.00	1.66 ± 0.02	0.14 ± 0.00
137	Agarospirol	Alcohol	1.3	958	1645	1646	1.77 ± 0.07	0.15 ± 0.00	*-*	*-*	*-*	*-*
138	Hinesol	Alcohol	1.3	974	1645	1645	1.25 ± 0.05	0.11 ± 0.00	*-*	*-*	*-*	*-*
139	*allo*-Aromandendrene epoxide	Epoxide	1.5	946	1649	1644	*-*	*-*	19.04 ± 0.30	1.42 ± 0.01	*-*	*-*
140	Pogostol	Alcohol	1.3	896	1649	1650	3.05 ± 0.09	0.26 ± 0.00	*-*	*-*	*-*	*-*
141	β-Eudesmol	Alcohol	1.3	933	1661	1656	30.97 ± 1.10	2.62 ± 0.04	*-*	*-*	*-*	*-*
142	14-hydroxy-(*Z*)-Caryophyllene	Alcohol	1.3	951	1662	1664	*-*	*-*	21.47 ± 0.46	1.85 ± 0.00	2.89 ± 0.06	0.23 ± 0.00
143	*neo*-Intermedeol	Alcohol	1.3	932	1662	1661	*-*	*-*	*-*	*-*	2.27 ± 0.06	0.18 ± 0.00
144	7-*epi*-α-Eudesmol	Alcohol	1.3	960	1667	1665	1.27 ± 0.05	0.11 ± 0.00	*-*	*-*	*-*	*-*
145	Intermedeol	Alcohol	1.3	967	1670	1668	*-*	*-*	1.95 ± 0.06	0.17 ± 0.00	1.03 ± 0.01	0.08 ± 0.00
146	Bulnesol	Alcohol	1.3	991	1670	1670	22.29 ± 0.76	1.89 ± 0.04	*-*	*-*	*-*	*-*
147	Khusinol	Alcohol	1.3	918	1677	1677	*-*	*-*	13.86 ± 0.26	1.19 ± 0.01	2.62 ± 0.05	0.21 ± 0.00
148	α-Bisabolol	Alcohol	1.3	983	1689	1688	24.80 ± 0.83	2.10 ± 0.04	4.85 ± 0.11	0.42 ± 0.00	29.22 ± 0.41	2.38 ± 0.00
149	Juniper camphor	Alcohol	1.3	928	1701	1696	*-*	*-*	0.79 ± 0.01	0.07 ± 0.00	*-*	*-*
150	Caryophyllene acetate isomer I	Ester	1.6	917	1704	1701	*-*	*-*	5.60 ± 0.12	0.39 ± 0.00	0.90 ± 0.02	0.06 ± 0.00
151	*iso*-Longifolol	Alcohol	1.3	933	1726	1727	*-*	*-*	*-*	*-*	0.24 ± 0.01	0.02 ± 0.00
152	Nootkatone	Ketone	1.3	974	1810	1806	*-*	*-*	*-*	*-*	*-*	*-*
153	Phytone	Ketone	1.3	991	1843	1841	*-*	*-*	2.08 ± 0.06	0.18 ± 0.00	0.65 ± 0.01	0.05 ± 0.00
154	*m*-Camphorene	Diterpene	1.0	979	1950	1946	0.29 ± 0.01	0.03 ± 0.00	0.27 ± 0.01	0.03 ± 0.00	0.36 ± 0.01	0.04 ± 0.00
155	*p*-Camphorene	Diterpene	1.0	983	1986	1984	0.11 ± 0.00	0.01 ± 0.00	0.34 ± 0.01	0.04 ± 0.00	0.21 ± 0.01	0.02 ± 0.00
156	Phytol	Alcohol	1.3	956	2111	2111	*-*	*-*	1.23 ± 0.03	0.14 ± 0.01	*-*	*-*
157	Cannabidivarin (CBDV)	Cannabinoid	1.0	897	2215	2216	*-*	*-*	0.74 ± 0.04	0.06 ± 0.00	*-*	*-*
158	Cannabicitran (CBT)	Cannabinoid	1.0	954	2281	2284	*-*	*-*	1.03 ± 0.05	0.08 ± 0.00	*-*	*-*
159	Cannabicyclol (CBL)	Cannabinoid	1.0	890	2373	2374	*-*	*-*	0.25 ± 0.02	0.02 ± 0.00	*-*	*-*
160	Cannabidiol (CBD)	Cannabinoid	1.0	983	2424	2421	2.24 ± 0.29	0.12 ± 0.01	20.06 ± 1.00	1.60 ± 0.01	4.67 ± 0.07	0.36 ± 0.00
161	Cannabichromene (CBC)	Cannabinoid	1.0	892	2433	2435	*-*	*-*	0.47 ± 0.06	0.04 ± 0.00	*-*	*-*
162	δ8-Tetrahydrocannabinol (Δ^8^-THC)	Cannabinoid	1.0	890	2501	2501	*-*	*-*	0.16 ± 0.01	0.01 ± 0.00	*-*	*-*
163	δ9-Tetrahydrocannabinol (Δ^9^-THC)	Cannabinoid	1.0	910	2525	2527	*-*	*-*	0.26 ± 0.01	0.02 ± 0.00	*-*	*-*
164	*n*-Heptacosane	Aliphatic	1.0	953	2701	2700	*-*	*-*	0.23 ± 0.01	0.03 ± 0.00	*-*	*-*
165	*n*-Nonacosane	Aliphatic	1.0	958	2902	2900	*-*	*-*	0.37 ± 0.01	0.04 ± 0.00	*-*	*-*
	*NOT IDENTIFIED*	-	-	-	-	-	15.07 ± 0.43	1.66 ± 0.02	75.61 ± 1.94	8.45 ± 0.02	37.05 ± 0.34	3.88 ± 0.02
	*TOTAL*	-	-	-	-	-	981.41 ± 22.53	100.00 ± 0.00	980.04 ± 22.72	100.00 ± 0.00	991.07 ± 8.77	100.00 ± 0.00
	*HYDROCARBON Compounds*	-	-	-	-	-	668.63 ± 14.51	73.39 ± 0.19	610.32 ± 13.90	68.04 ± 0.04	796.68 ± 7.16	83.86 ± 0.01
	*Monoterpenes*	-	-	-	-	-	424.22 ± 3.34	46.48 ± 0.27	142.26 ± 3.05	15.68 ± 0.06	119.17 ± 5.25	12.27 ± 0.08
	*Sesquiterpenes*	-	-	-	-	-	244.00 ± 11.25	26.87 ± 0.08	466.57 ± 10.80	52.19 ± 0.02	676.94 ± 8.24	71.53 ± 0.07
	*Diterpenes*	-	-	-	-	-	0.40 ± 0.01	0.04 ± 0.00	0.61 ± 0.02	0.07 ± 0.00	0.57 ± 0.01	0.06 ± 0.00
	*Aliphatic*	-	-	-	-	-	0.00 ± 0.00	0.00 ± 0.00	0.89 ± 0.03	0.10 ± 0.00	0.00 ± 0.00	0.00 ± 0.00
	*OXIGENATED Compounds*	-	-	-	-	-	297.72 ± 7.68	24.95 ± 0.19	294.11 ± 6.88	23.51 ± 0.06	157.34 ± 2.86	12.26 ± 0.02
	*Aldehydes*	-	-	-	-	-	1.01 ± 0.08	0.08 ± 0.01	1.19 ± 0.01	0.10 ± 0.00	3.23 ± 0.16	0.26 ± 0.00
	*Alcohols*	-	-	-	-	-	280.45 ± 6.87	23.71 ± 0.18	123.30 ± 2.56	10.62 ± 0.02	111.55 ± 2.76	8.94 ± 0.01
	*Esters*	-	-	-	-	-	3.02 ± 0.05	0.21 ± 0.00	8.04 ± 0.15	0.60 ± 0.00	1.95 ± 0.01	0.13 ± 0.00
	*Ketones*	-	-	-	-	-	2.02 ± 0.03	0.17 ± 0.00	3.50 ± 0.07	0.30 ± 0.00	4.81 ± 0.15	0.38 ± 0.00
	*Epoxides*	-	-	-	-	-	8.98 ± 0.39	0.66 ± 0.01	135.13 ± 2.91	10.08 ± 0.02	31.13 ± 0.37	2.19 ± 0.00
	*Cannabinoids*	-	-	-	-	-	2.24 ± 0.29	0.12 ± 0.01	22.95 ± 1.17	1.83 ± 0.01	4.67 ± 0.07	0.36 ± 0.00
	*Distillation Yield*	-	-	-	-	-	0.200		0.016		0.109	

**Table 3:** Measurements of response factors (RFs) for different cannabinoid compounds.

Compounds	Rep. 1	Rep. 2	Rep. 3	Mean ± SD	RF
Cannabidivarin (CBDV)	0.992	0.998	0.992	0.994 ± 0.003	1.0
Cannabicitran (CBT)	0.917	0.966	0.974	0.952 ± 0.031	1.0
Cannabicyclol (CBL)	0.982	0.980	0.978	0.980 ± 0.002	1.0
Cannabidiol (CBD)	0.981	0.981	0.979	0.980 ± 0.002	1.0
Cannabichromene (CBC)	1.050	1.050	1.042	1.047 ± 0.002	1.0
δ8-Tetrahydrocannabinol (Δ^8^-THC)	1.062	0.996	0.997	1.018 ± 0.038	1.0
δ9-Tetrahydrocannabinol (Δ^9^-THC)	1.019	0.959	0.956	0.978 ± 0.036	1.0

**Table 4:** Enantiomeric distribution of selected components in cannabis EOs from different cultivars. Abbreviations: match: database spectral similarity; enantio-LRI_exp_: chiral experimental LRI; enantio-LRI_ref_: chiral reference LRI; MS signal: mass signal monitored.

Enantiomer	Match	Enantio-LRI_exp_	Enantio-LRI_ref_	MS Signal	Kompolti		Futura 75		Carmagnola	Felina 32	Finola
					Fresh	Dried	Fresh	Dried	Fresh	Dried	Fresh
(*R*)-(−)-a-Pinene	941	1021	1020	TIC	4.03	8.72	5.91	6.01	94.02	8.31	12.11
(*S*)-(+)-a-Pinene	994	1026	1025		95.97	91.28	94.09	93.99	5.98	91.69	87.89
(*S*)-(−)-Camphene	974	1060	1061	TIC	42.10	42.12	37.89	40.48	95.55	29.82	59.71
(*R*)-(+)-Camphene	940	1066	1067		57.90	57.88	62.11	59.52	4.45	70.18	40.29
(*R*)-(+)-β-Pinene	984	1080	1083	TIC	83.70	79.95	85.61	83.51	97.34	83.15	68.07
(*S*)-(−)-β-Pinene	956	1085	1086		16.30	20.05	16.49	16.49	2.66	16.85	31.93
(*S*)-(−)-Limonene	995	1100	1102	TIC	86.45	92.30	-	73.22	94.02	83.15	91.90
(*R*)-(+)-Limonene	991	1105	1106		13.55	7.70	-	26.78	5.98	16.85	8.10
(*R*)-(−)-Linalool	965	1260	1261	TIC	5.79	27.60	-	21.78	2.20	32.23	20.5
(*S*)-(+)-Linalool	979	1263	1264		94.21	72.40	-	78.22	97.80	67.77	79.5
(*R*)-(+)-Fenchyl alcohol	992	1357	1358	TIC	98.24	92.30	-	94.83	99.31	87.16	99.38
(*S*)-(−)-Fenchyl alcohol	932	1360	1362		1.76	7.70	-	5.17	0.69	12.84	0.62
(*S*)-(−)-Borneol	977	1439	1439	95 *m/z*	83.11	66.84	63.83	73.44	97.34	62.18	94.24
(*R*)-(+)-Borneol	971	1447	1450		16.89	33.16	36.17	26.56	2.66	37.82	5.76
(*R*)-(−)-(*E*)-Caryophyllene	993	1501	1504	133 *m/z*	100.00	100.00	100.00	100.00	100.00	100.00	100.00
(*R*)-(−)-(E)-Nerolidol	947	1715	1716	69 *m/z*	4.98	0.56	2.63	1.50	0.75	0.76	2.76
(*S*)-(+)-(E)-Nerolidol	994	1718	1719		95.02	99.44	97.37	98.50	99.25	99.24	97.24
(*S*)-(+)-Caryophyllene oxide	987	1739	1739	79 *m/z*	-	1.90	0.98	8.57	8.50	0.99	1.78
(*R*)-(−)-Caryophyllene oxide	993	1746	1747		-	98.10	99.02	91.43	91.50	99.01	98.22

In the fresh Futura 75 sample, the enantio-distribution of limonene was not determined due to a coelution of (+)-limonene with a component.

## Supplementary Materials

The following are available online, Table S1: List of volatile compounds detected in dried inflorescences cultivar Futura 75 distilled at different 37 time periods at 700 W: 10 min, 20 min, 30 min and 40 min. Total amounts are expressed in mg g^−1^. The 38 compounds are also grouped on the base of chemical classes including monoterpene, sesquiterpene, oxygenated 39 compounds and cannabinoids. The cannabis EO yields are also reported.

## Sample Availability

Samples of the compounds are not available from the authors.
