# Supplementary material for: Development of a Novel Microwave Distillation Technique for the Isolation of Cannabis sativa L. Essential Oil and Gas Chromatography Analyses for the Comprehensive Characterization of Terpenes and Terpenoids, Including Their Enantio-Distribution
Source: Molecules. 2021 Mar 13;26(6):1588. doi: 10.3390/molecules26061588 (PMC8000122; doi:10.3390/molecules26061588)
Supplement: Supplementary file 1 [file molecules-26-01588-s001.pdf]

molecules

Article

Development of a novel microwave distillation technique for the isolation of *Cannabis Sativa L.* essential oil and gas chromatography analyses for the comprehensive characterization of terpenes and terpenoids, including their enantio-distribution.

Giuseppe Micalizzi [1]*, Filippo Alibrando [1], Federica Vento [1], Emanuela Trovato [1], Mariosimone Zoccali [2], Paolo Guarnaccia[3], Paola Dugo [1,4,5] and Luigi Mondello[1,4,5]

[1] Chromaleont s.r.l., c/o Department of Chemical, Biological, Pharmaceutical and Environmental Sciences, University of Messina, Viale Palatucci, Polo Universitario Annunziata - 98168 Messina, Italy; giuseppe.micalizzi@chromaleont.it (G.M.); filippo.alibrando@chromaleont.it (F.A.); federica.vento@chromaleont.it (F.V.); emanuela.trovato@chromaleont.it (E.T.)

[2] Department of Mathematical and Computer Science, Physical Sciences and Earth Sciences, University of Messina, Viale Ferdinando Stagno d'Alcontres 31 - 98166 Messina, Italy; mzoccali@unime.it (M.Z.)

[3] Department of Agriculture, Food and Environment (Di3A), University of Catania, via Valdisavoia, 5 – 95123 Catania, Italy; paolo.guarnaccia@unict.it (P.G.)

[4] Department of Chemical, Biological, Pharmaceutical and Environmental Sciences, University of Messina, Viale Palatucci, Polo Universitario Annunziata - 98168 Messina, Italy.

[5] BeSep s.r.l., c/o Department of Chemical, Biological, Pharmaceutical and Environmental Sciences, University of Messina, Viale Palatucci, Polo Universitario Annunziata - 98168 Messina, Italy.; pdugo@unime.it (P.D.); lmondello@unime.it (L.M.)

* Correspondence: giuseppe.micalizzi@chromaleont.it; Tel.: +39 3401647106

Table S1. List of volatile compounds detected in dried inflorescences cultivar Futura 75 distilled at different time periods at 700 W: 10 min, 20 min, 30 min and 40 min. Total amounts are expressed in mg g^{-1}. The compounds are also grouped on the base of chemical classes including monoterpene, sesquiterpene, oxygenated compounds and cannabinoids. The cannabis EO yields are also reported.

Compounds	10 min	20 min	30 min	40 min
Hashishene	0.52 ± 0.01	0.46 ± 0.01	0.39 ± 0.00	0.52 ± 0.02
α-Thujene	0.40 ± 0.02	0.33 ± 0.01	0.25 ± 0.02	0.39 ± 0.03
α-Pinene	146.36 ± 5.87	113.79 ± 3.59	91.91 ± 2.19	132.26 ± 6.82
α-Fenchene	0.19 ± 0.00	0.15 ± 0.01	0.13 ± 0.00	0.17 ± 0.01
Camphene	2.37 ± 0.08	1.91 ± 0.04	1.59 ± 0.05	2.19 ± 0.14
Sabinene	0.30 ± 0.01	0.27 ± 0.02	0.24 ± 0.01	0.32 ± 0.01
β-Pinene	26.85 ± 0.79	21.94 ± 0.48	18.33 ± 0.18	24.56 ± 0.90
6-methyl-Hept-5-en-2-one	0.19 ± 0.02	0.16 ± 0.01	0.16 ± 0.00	0.16 ± 0.01
Myrcene	58.67 ± 2.24	59.17 ± 1.65	56.56 ± 1.03	61.21 ± 2.59
α-Phellandrene	1.09 ± 0.07	1.12 ± 0.07	1.02 ± 0.03	1.33 ± 0.07
δ3-Carene	28.76 ± 1.01	28.72 ± 0.70	21.98 ± 0.23	25.40 ± 0.94
α-Terpinene	1.39 ± 0.07	1.35 ± 0.07	1.28 ± 0.02	1.59 ± 0.09
p-Cymene	0.69 ± 0.03	0.89 ± 0.14	0.65 ± 0.01	0.77 ± 0.02
Limonene	10.19 ± 0.32	9.25 ± 0.20	9.07 ± 0.10	10.00 ± 0.33

β-Phellandrene	1.93 ± 0.03	2.06 ± 0.06	2.66 ± 1.52	2.27 ± 0.05
Eucalyptol	15.57 ± 0.37	12.89 ± 0.22	10.35 ± 1.33	11.67 ± 0.25
(E)-, β-Ocimene	51.94 ± 1.76	49.14 ± 2.55	41.44 ± 0.56	50.76 ± 2.05
γ-Terpinene	2.72 ± 0.07	2.48 ± 0.15	2.46 ± 0.03	2.61 ± 0.09
(Z)-Sabinene hydrate	1.12 ± 0.12	0.93 ± 0.13	0.79 ± 0.07	0.78 ± 0.05
Terpinolene	12.03 ± 0.30	13.94 ± 0.52	13.01 ± 0.14	15.15 ± 0.46
p-Cymenene	0.36 ± 0.01	0.42 ± 0.02	0.38 ± 0.01	0.46 ± 0.02
Linalool	3.01 ± 0.09	2.85 ± 0.13	2.34 ± 0.09	2.51 ± 0.02
(E)-Sabinene hydrate	0.38 ± 0.15	0.41 ± 0.17	0.35 ± 0.04	0.35 ± 0.12
n-Nonanal	0.96 ± 0.04	0.92 ± 0.06	0.95 ± 0.04	1.04 ± 0.11
Fenchyl alcohol	1.25 ± 0.03	1.10 ± 0.04	1.04 ± 0.04	0.99 ± 0.01
allo-Ocim-(4E,6Z)-ene	1.62 ± 0.27	1.43 ± 0.11	1.42 ± 0.29	1.39 ± 0.02
Borneol	0.78 ± 0.03	0.76 ± 0.07	0.73 ± 0.05	0.72 ± 0.01
Terpinen-4-ol	0.89 ± 0.02	0.86 ± 0.09	0.77 ± 0.04	0.75 ± 0.00
α-Terpineol	1.08 ± 0.01	0.67 ± 0.59	1.01 ± 0.06	1.02 ± 0.03
α-Ylangene	1.64 ± 0.05	1.81 ± 0.07	1.89 ± 0.06	1.61 ± 0.01
7-*epi*-Sesquithujene	0.24 ± 0.07	0.25 ± 0.09	0.28 ± 0.08	0.37 ± 0.16
α-Funebrene	0.22 ± 0.01	0.22 ± 0.01	0.26 ± 0.02	0.28 ± 0.14
(Z)-Caryophyllene	2.16 ± 0.06	2.46 ± 0.12	2.72 ± 0.11	1.98 ± 0.11
α -, (Z)-Bergamotene	2.13 ± 0.06	1.91 ± 0.06	2.48 ± 0.10	1.81 ± 0.12
(E)-Caryophyllene	211.02 ± 5.12	238.18 ± 3.36	252.45 ± 4.23	205.86 ± 3.11
α-, (E)-Bergamotene	15.53 ± 0.35	14.35 ± 0.37	17.97 ± 0.51	13.23 ± 0.07
α-Guaiene	1.05 ± 0.05	1.31 ± 0.04	1.24 ± 0.05	1.70 ± 0.07
Guaia-6,9-diene	1.26 ± 0.11	1.41 ± 0.13	1.62 ± 0.03	1.37 ± 0.06
(E)-Geranylacetone	0.88 ± 0.04	1.00 ± 0.03	1.25 ± 0.05	1.18 ± 0.14
(E)-, β-Farnesene	18.12 ± 0.44	16.29 ± 0.42	20.99 ± 0.74	15.50 ± 0.09
α-Humulene	59.41 ± 1.22	67.41 ± 1.02	72.46 ± 1.05	60.81 ± 0.75
9-*epi*-(E)-Caryophyllene	3.54 ± 0.09	4.58 ± 0.10	4.85 ± 0.13	4.59 ± 0.16
Drima-7,9(11)-diene	0.74 ± 0.07	0.88 ± 0.07	0.97 ± 0.13	0.93 ± 0.17
Selina-4,11-diene	1.04 ± 0.09	1.36 ± 0.18	1.38 ± 0.04	1.25 ± 0.10
γ-Gurjunene	1.51 ± 0.09	1.36 ± 0.12	1.69 ± 0.02	1.51 ± 0.09
Aristolochene	7.08 ± 0.45	7.88 ± 0.29	8.47 ± 0.69	7.11 ± 0.28
Eremophilene	2.03 ± 0.35	2.16 ± 0.30	2.17 ± 0.45	2.25 ± 0.45
β-Selinene	16.91 ± 0.43	19.31 ± 0.52	20.62 ± 0.67	17.85 ± 0.24
Valencene	3.27 ± 0.13	3.69 ± 0.10	3.93 ± 0.04	3.49 ± 0.24
α-Selinene	13.24 ± 0.32	14.97 ± 0.38	16.12 ± 0.62	14.29 ± 0.10
(E,E)-, α-Farnesene	3.45 ± 0.08	3.35 ± 0.10	4.09 ± 0.05	3.61 ± 0.04
β-Bisabolene	6.42 ± 0.42	7.26 ± 0.48	8.16 ± 0.14	7.35 ± 0.47
β-Sesquiphellandrene	0.67 ± 0.02	0.63 ± 0.01	0.76 ± 0.01	0.79 ± 0.06
Selina-4(15),7(11)-diene	24.23 ± 0.51	24.44 ± 0.51	25.58 ± 0.73	29.87 ± 0.23
Selina-3,7(11)-diene	21.81 ± 0.44	19.58 ± 0.44	20.96 ± 0.66	24.49 ± 0.24
(E)-Nerolidol	7.14 ± 0.38	8.12 ± 0.39	9.73 ± 0.64	11.49 ± 0.54
Caryophyllene oxide	18.22 ± 3.35	22.53 ± 4.10	28.06 ± 4.15	31.11 ± 3.52
Humulene epoxide II	6.32 ± 0.45	7.79 ± 0.68	9.58 ± 0.58	11.10 ± 0.60
Intermedeol	3.39 ± 0.17	4.03 ± 0.12	5.25 ± 0.42	5.69 ± 0.49
Caryophylla-4(12),8(13)-dien-5-alpha-ol	2.36 ± 0.17	2.83 ± 0.19	4.03 ± 0.02	3.88 ± 0.07
α-Bisabolol	7.42 ± 0.40	9.81 ± 0.49	11.99 ± 0.78	15.89 ± 0.74
Phytone	1.16 ± 0.06	1.41 ± 0.08	2.01 ± 0.13	2.44 ± 0.14
m-Camphorene	0.20 ± 0.10	0.29 ± 0.14	0.32 ± 0.13	0.41 ± 0.26

p-Camphorene	0.18 ± 0.03	0.23 ± 0.05	0.42 ± 0.20	0.40 ± 0.00
Phytol	0.57 ± 0.30	0.87 ± 0.49	1.42 ± 0.93	2.02 ± 0.00
Cannabidivarin (CBDV)	0.19 ± 0.03	0.20 ± 0.00	0.39 ± 0.07	0.47 ± 0.00
Cannabicitran (CBT)	0.67 ± 0.10	1.09 ± 0.02	1.17 ± 0.22	1.40 ± 0.00
Cannabicyclol (CBL)	-	-	0.11 ± 0.02	0.11 ± 0.00
Cannabidiol (CBD)	7.09 ± 0.57	6.81 ± 0.17	13.52 ± 2.35	14.37 ± 0.05
Cannabichromene (CBC)	0.20 ± 0.03	0.28 ± 0.03	0.35 ± 0.04	0.44 ± 0.02
n-Pentacosane	-	-	0.13 ± 0.03	0.11 ± 0.00
δ9-Tetrahydrocannabinol (Δ^9-THC)	0.19 ± 0.03	0.17 ± 0.00	0.37 ± 0.05	0.47 ± 0.00
n-Heptacosane	0.14 ± 0.03	0.22 ± 0.01	0.20 ± 0.06	0.21 ± 0.02
n-Nonacosane	0.14 ± 0.05	0.21 ± 0.01	0.21 ± 0.10	0.19 ± 0.02
NOT IDENTIFIED	77.46 ± 6.18	92.36 ± 6.58	84.63 ± 21.75	74.72 ± 8.40
TOTAL	926.22 ± 17.30	944.78 ± 15.09	952.47 ± 24.12	954.00 ± 7.00
Monoterpenes	348.38 ± 12.41	308.81 ± 9.69	275.92 ± 2.98	345.80 ± 14.78
Sesquiterpenes	418.70 ± 8.72	458.08 ± 6.94	494.10 ± 5.91	425.06 ± 4.66
Oxygenated Compounds	72.69 ± 5.12	79.94 ± 7.28	91.80 ± 7.33	104.25 ± 6.36
Cannabinoids	8.33 ± 0.69	8.55 ± 1.57	15.91 ± 2.69	16.45 ± 1.39
Distillation Yield	0.019 %	0.025 %	0.024 %	0.035 %
